# Extracellularly Activatable Conjugates of RGD Peptidomimetics and Cryptophycin for α_V_β_3_‐Targeted Cancer Therapy

**DOI:** 10.1002/chem.71270

**Published:** 2026-06-18

**Authors:** Dominic Seißenschmidt, Henriette Hartung, Jonah Kammer, Norbert Sewald

**Affiliations:** ^1^ Organic and Bioorganic Chemistry Faculty of Chemistry Bielefeld University Bielefeld Germany

**Keywords:** antitumor agents, cryptophycin, drug delivery, neutrophil elastase, RGD peptidomimetic

## Abstract

Small molecule‐drug conjugates (SMDCs) represent a promising approach in targeted cancer therapy, combining defined tumor‐targeting ligands with powerful payloads. Here, we report the design, synthesis, and biological evaluation of integrin α_V_β_3_‐targeting SMDCs based on a highly potent cryptophycin payload and a neutrophil elastase cleavable NPV‐PABC linker. A series of stereochemically defined RGD peptidomimetics, linker epimers, and corresponding SMDCs were synthesized, providing additional negative controls for both targeting and enzymatic cleavage. The resulting conjugates remained stable in human plasma, preserved integrin‐binding affinity in enzyme‐linked immunosorbent assays (ELISA), and released the payload rapidly upon exposure to human neutrophil elastase depending on the stereochemistry of the linker. In vitro cytotoxicity studies in multiple cancer cell lines demonstrated that enzymatic cleavage restored cryptophycin activity to near free‐drug levels, yielding picomolar potency.

## Graphical abstract

Integrin α_V_β_3_‐targeting small‐molecule drug conjugates (SMDCs) were synthesized by conjugating a cryptophycin payload through a neutrophil elastase‐cleavable NPV‐PABC linker. RGD peptidomimetic and linker epimers served as controls for targeting and enzymatic cleavage, and the resulting conjugates displayed subnanomolar cytotoxicity in vitro.

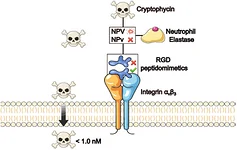

## 1 Introduction

In 2023, cancer was the second leading cause of mortality, accounting for 21% of all deaths, with close to 13 million cases across OECD (Organization for Economic Co‐operation and Development) countries [1]. Consequently, there is an urgent need for the development of anticancer medications. However, patients treated with traditional chemotherapy suffer from serious side effects like nausea, vomiting, fatigue, and hair loss [2], because commonly used chemotherapeutic agents—such as antimetabolites, alkylating agents, microtubule‐targeting agents, or topoisomerase inhibitors—are administered in a non‐targeted manner and, in addition to cancer cells, also effect other fast‐proliferating cells, for example, those of the bone marrow, gastrointestinal tract, and hair follicles [3]. Therefore, current research focuses on targeted cancer therapy, utilizing antibody–drug conjugates (ADCs) [4] and small molecule‐drug conjugates (SMDCs) [5].

In this field of research, we are particularly interested in cryptophycins, a promising class of payloads, which are macrocyclic depsipeptides with remarkable cytotoxicity in the picomolar range, even against multidrug‐resistant (MDR) cell lines, and that act as microtubule destabilizers [6]. However, the use of Cryptophycin‐52 (LY355703) as standalone cytostatic drug was discontinued, because of neurotoxic side effects observed in clinical studies conducted by scientists at Eli Lilly [7, 8], making conjugation unavoidable. Since the naturally occurring cryptophycin‐1 and its synthetic analogue cryptophycin‐52 lack a functional group for linker attachment various modifications introducing different functional groups in all four units (A to D) [9] of the cryptophycin scaffold have been investigated. Based on cryo‐electron microscopy and crystallographic studies of a cryptophycin–tubulin complex, unit D analogues were identified as the most suitable [10, 11]. Intensive structure–activity relationship (SAR) studies including unit D derivatives containing amino, hydroxy, carboxy, and sulfur‐based functional groups revealed that N‐alkylated 2,3‐diaminopropanoate (Dap)‐derived cryptophycins are the most potent derivatives, making them highly promising candidates as cytotoxic payloads [12].

When designing an SMDC, one can choose from a variety of small‐molecule and peptide ligands and their corresponding targets that are currently being explored, for example, folate (folate receptor FRα) [13], arginine–glycine–aspartate (RGD) peptides (integrins) [14], oncoFAP (fibroblast activation protein) [15], DUPA (prostate‐specific membrane antigen) [16], as well as different payload‐release strategies (cathepsin [17], neutrophil elastase [18], hydrolysis [19], reduction [19], β‐glucuronidase [20], sulfatase [21], phosphatase [22], legumain [23], and matrix metalloproteinase‐2 [24]).

For initial conjugation studies, the Dap(Me)‐derived cryptophycin (cytotoxicity IC_50_ (KB‐3‐1): 8.71 pM) [12] was used in folate receptor‐targeting cathepsin B‐cleavable SMDCs, linked via a triazole either through copper‐catalyzed azide‐alkyne cycloaddition (CuAAC) or strain‐promoted azide‐alkyne cycloaddition (SPAAC) [25]. However, the resulting conjugates did not show any selectivity between folate receptor‐positive KB‐3‐1 and folate receptor‐negative HEK293 cells in cytotoxicity assays, exhibited a substantial loss of activity compared to the unconjugated Dap(Me)‐derived cryptophycin, and showed inferior cleavability compared to literature‐reported SMDCs [26]. Based on these results, we envisioned a different approach for conjugation of the Dap(Me)‐derived cryptophycin.

## 2 Results and Discussion

We aimed at replacing the folate ligand with an RGD peptidomimetic previously developed by Paulus, derived from l‐tyrosine and exhibiting a high targeting index of 134 when used in a GKGEVA‐PABC‐MMAE conjugate [27]. In addition, we sought to establish an appropriate negative control for the RGD peptidomimetic by altering the stereochemistry of the l‐tyrosine scaffold, which is expected to result in a significant loss of affinity toward the α**_V_** β_3_ integrin, according to the literature [28, 29]. Indeed, control conjugates employing c(RGDfK) and c(RADfK) peptides as homing devices showed targeting indices of 0.2 and similar cytotoxicity [27]. To overcome poor cleavability of previously described cathepsin B‐cleavable SMDCs [25], we further envisioned utilizing neutrophil elastase, a serine protease, as the cleavage‐inducing enzyme, which is overexpressed in the tumor microenvironment and enables extracellular cleavage of an Asn–Pro–Val (NPV) tripeptide linker within minutes, similar to reports by Lerchen, while the stereochemically altered NPv tripeptide remains unaffected by neutrophil elastase [28]. Using the commonly employed self‐immolative para‐aminobenzyl carbamate (PABC) spacer for traceless release of the cytotoxic payload and click chemistry for ligand assembly, while also altering the stereochemistry of the linker and RGD‐peptidomimetic, would result in an SMDC and its epimers being negative controls for targeting and cleavability (Figure 1).

**FIGURE 1:**
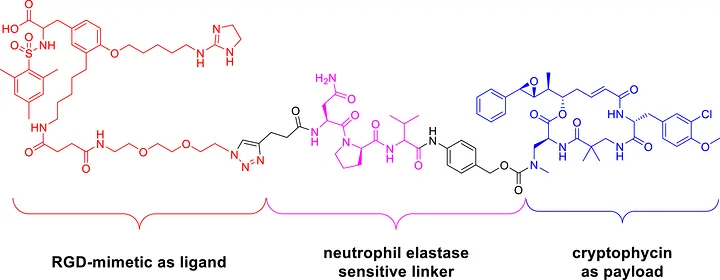
Structure of the envisioned neutrophil elastase cleavable, integrin α_V_β_3_ targeting RGD‐peptidomimetic cryptophycin conjugates displaying different stereochemistry in linker and peptidomimetic as negative controls.

### 2.1 Synthesis of RGD Mimetic Epimers

The synthesis of the various RGD peptidomimetics (Figure 1) was adapted and modified based on the methodology described by Paulus [27]. l‐ or d‐Tyrosine was iodinated in aqueous ammonia by slow addition of iodine under cooling to prevent formation of the explosive nitrogen triiodide [30]. Diiodinated tyrosine was observed, but the undesired product was successfully separated during chromatographic purification of compounds **3a–b**, which were subsequently synthesized by protection of the C‑terminus as a methyl ester using thionyl chloride in methanol, followed by Boc protection of the amino group (Scheme 1). 5‑Aminopentan‐1‐ol (**4**) was Cbz protected and converted into alkyl iodide **6** via a modified Appel reaction [31] (Scheme 1). Attempts to reproduce the Negishi cross‐coupling reported by Paulus, involving separate formation of the alkylzinc iodide using untreated zinc powder and trimethylsilyl chloride, followed by addition to a mixture of aryl iodides **3a–b**, bis(dibenzylideneacetone)palladium(0) (Pd^0^(dba)_2_) and tri(*o*‑tolyl)phosphine (P(*o*‐tolyl)_3_), led to incomplete conversion of aryl iodide, as monitored by LCMS, resulting in poor yields. To overcome this, we employed a system established by Cerveson [32], designed for peptide Negishi cross‐coupling under mild conditions. Using preactivated zinc dust, dichlorobis(amphos)palladium(II) (Pd(amphos)_2_Cl_2_), and omitting preformation of the alkylzinc intermediate led to full conversion of aryl iodides **3a–b** and increased yields up to 64 % (Lit.: 31 %) [27] (Scheme 1). Minor dehalogenation of the alkylzinc iodide, presumably due to residual water, was still detected by LCMS. Notably, upscaling the Negishi cross‐coupling from typically 50 µmol to 1.5 mmol with respect to the aryl iodide proceeded smoothly without further need for optimization.

**SCHEME 1:**
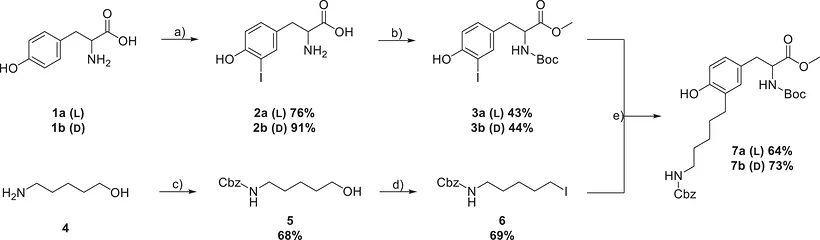
Synthesis of Negishi products **7a–b**. (a) I_2_, NH_3 _(25 % aq.), EtOH, ‐10°C, 1 h; (b) 1. SOCl_2_, MeOH, 65°C, 1 h; 2. Boc_2_O, NaOH, THF/H_2_O (1:1), RT, 18 h; (c) CbzCl, K_2_CO_3_, acetone/H_2_O (1:1), 0°C > RT, 18 h; (d) I_2_, PPh_3_, imidazole, 0°C, 1 h → RT, 18 h; (e) Zn, Pd(amphos)_2_Cl_2_, DMF, 40°C, 18 h. a = l‐configured and b = d‐configured.

Amino alcohol **4** was Alloc‐protected to ensure orthogonality between the alkyl amines in **9a–b** used as connectors to the tyrosine scaffold (Scheme 2). Alkylaryl ethers **9a–b** were synthesized via a Mitsunobu reaction employing 1,1‑(azodicarbonyl)dipiperidine (ADDP) and tributylphosphine (TBP), as the commonly used diethyl azodicarboxylate (DEAD) and triphenyl phosphine (TPP) system led to incomplete conversion of aryl‐modified tyrosines **7a–b** and prolonged reaction times. DEAD‐TPP Mitsunobu reactions typically perform well only with substrates having a pK_a_ < 11 [33]. In contrast, ADDP is more readily protonated by less acidic substrates, facilitating improved reaction efficiency. Due to its lower reactivity as a Michael acceptor compared to DEAD, ADDP requires pairing with more nucleophilic phosphines such as TBP to achieve optimal conversion [34]. Using the ADDP‐TBP system, alkylaryl ethers **9a‐b** were obtained in fair to very good yields within one hour. Subsequent one‐pot transformations, including acidolytic Boc deprotection, installation of the mesitylene sulfonyl moiety via nucleophilic substitution, Alloc deprotection using tetrakis(triphenylphosphine)palladium and excess morpholine as scavenger, and introduction of the guanidino group using 2‐methylthio‐2‐imidazoline, afforded RGD mimetic precursors **10a–b** in good to very good yields (Scheme 2).

**SCHEME 2:**
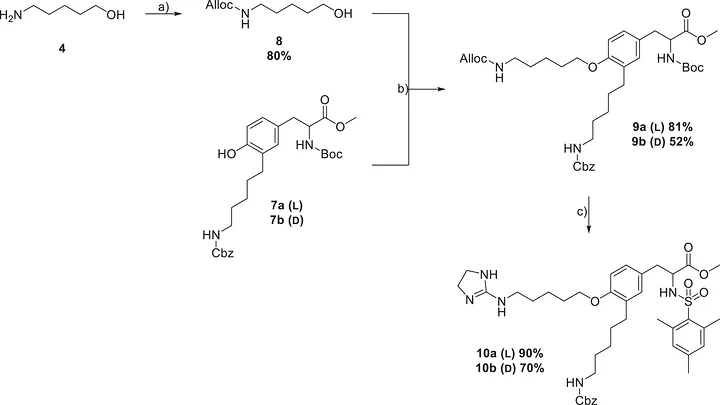
Synthesis of RGD mimetic precursors **10a–b**. (a) AllocCl, NaHCO_3_, THF/H_2_O (1:1), 0°C → RT, 18 h; (b) ADDP, P(n‐Bu)_3_, THF, 60°C, 1 h; (c) 1. HCl in 1,4‐dioxane, DCM, RT, 2 h; 2. mesitylene sulfochloride, DIPEA, DCM, DMF, RT, 18 h; 3. Pd(PPh_3_)_4_, morpholine, DCM, RT, 1 h; 4. methylthioimidazol, MeOH/Et_3_N (1:1), 80°C, 4 h. a = l‐configured and b = d‐configured.

Synthesis of the polyethylene glycol (PEG)‐ylated azide linker **13** was done as reported by Martin [35]. Tosylation of triethylene glycol, followed by introduction of the azide moiety using sodium azide and a subsequent Staudinger reaction, afforded amino azide **12**, which was then reacted with succinic anhydride (Scheme 3). Precursors **14a‐b** were obtained via Cbz deprotection of alkylaryl ethers **10a–b**, followed by coupling with PEGylated azide linker **13** using *O*‐(7‐azabenzotriazol‐1‐yl)‐N,N,N′,N′‐tetramethyluronium hexafluorophosphate (HATU). This step requires careful control, as HATU can also react with the guanidino moiety, leading to double coupling, which can be mitigated by using appropriate equivalents or less activating reagents, such as benzotriazol‐1‐yloxytripyrrolidinophosphonium hexafluorophosphate (PyBOP) [36]. Saponification of precursors **14a‑b** yielded the final conjugable RGD peptidomimetics **15a–b** (Scheme 3). Affinity toward integrin α_V_β_3_ was determined using an ELISA‐like assay (Table 1). As expected, the enantiomeric mimetic **15b** exhibited a 32‐fold lower integrin affinity compared to **15a**, providing a suitable negative control.

**SCHEME 3:**
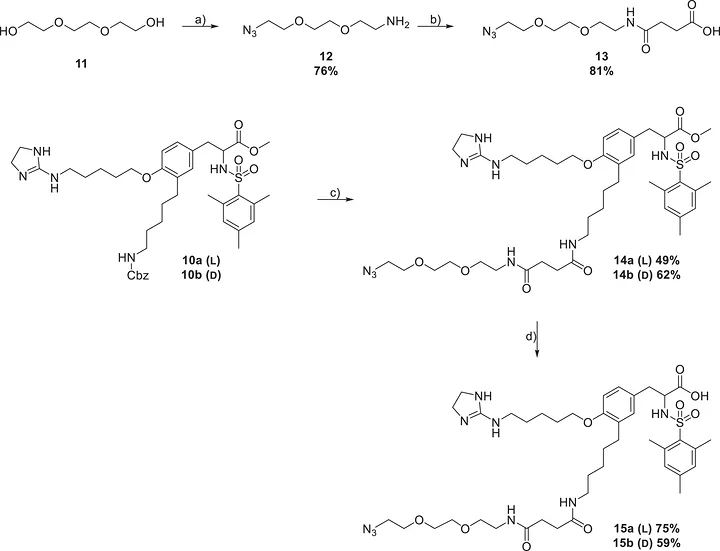
Synthesis of conjugable RGD mimetics **15a–b** and succinoyl‐PEG_2_ azide **13**. (a) 1. TosylCl, KOH, DCM, 0°C, 3 h; 2. NaN_3_, TBAI, DMF, 80°C, 18 h; 3. PPh_3_, Et_2_O, 1 M HCl (aq.), THF, RT, 18 h; (b) Succinic anhydride, ACN, RT, 18 h; (c) 1. Pd/C, H_2_ (800 mbar), MeOH, RT, 1 h; 2. **13**, HATU, DIPEA, DMF, RT, 2 h. a = l‐configured and b = d‐configured.

**TABLE 1:** Affinities of RGD peptidomimetics **15a–b** and SMDCs **21–1** (positive control), **21–3** (negative control) to integrin α_V_β_3_ determined in an ELISA‐type assay. Cilengitide (IC_50_: 6.34 ± 1.32 nM) was used as reference. IC_50_ values were obtained by sigmoidal dose response fitting of the mean values of 3–5 technical replicates. Error values represent the standard error of the fitted parameter.

Compound	Integrin α_V_β_3_ IC_50_ [nM]
**15a**	150 ± 24.1
**15b**	4745 ± 265
**21–1**	62.6 ± 4.27
**21–3**	2126 ± 254

### 2.2 Linker Attachment to Cryptophycin and Final SMDC Synthesis

In agreement with previously reported SMDCs, both a neutrophil elastase‐cleavable linker and its non‐cleavable derivative were synthesized [28]. Neutrophil elastase is overexpressed in the tumor microenvironment; therefore, extracellular payload release is expected for the cleavable construct. However, integrin‐mediated endocytosis, triggered by the RGD peptidomimetic, could potentially limit extracellular cleavage by promoting internalization of the SMDC. To date, internalization studies with this specific RGD mimetic have not been reported. Nevertheless, indirect evidence suggests that cellular uptake is likely. Cathepsin B‐cleavable SMDCs based on mimetic **15a** exhibit high cytotoxicity, which is consistent with an internalization‐dependent mechanism [27]. In addition, integrin binding was confirmed using an ELISA‐like assay (Table 1), supporting receptor engagement as a prerequisite for either uptake or extracellular retention. Taken together, these observations support two possible payload‐release mechanisms. In the first scenario, the SMDC remains extracellular, where linker cleavage by neutrophil elastase releases the payload, which then enters the cell by passive diffusion. In the second scenario, the SMDC is internalized into lysosomes, where proteases such as cathepsins, tripeptidyl peptidase I (TPP1), and dipeptidyl peptidase II (DPP‑II) are active [37]. Although specific cleavage of the NPV linker by lysosomal proteases has not been reported, lysosomal enzymes including cathepsins and legumain are known to cleave a broad range of peptide linkers [38, 39]. This suggests that lysosomal payload release remains a possible mechanism. Peptides **16a–b** were synthesized from Fmoc‐l/d‐Val‐OH using solid‐phase peptide synthesis following the Fmoc‐strategy, with Oxyma and *N,N’*‐diisopropylcarbodiimide (DIC) as coupling reagents. The C‑terminus was functionalized with *p*‑aminobenzyl alcohol (PAB) using *N*‐ethoxycarbonyl‐2‐ethoxy‐1,2‐dihydroquinoline (EEDQ), and the resulting benzyl alcohols were activated as *p*‐nitrophenyl carbonates **18a–b**. Cryptophycin **19** was then introduced under basic conditions, affording carbamates **20a–b** in good yields. Final conjugation between mimetics **15a–b** and linker‐attached cryptophycins **20a–b** was accomplished via CuAAC (Scheme 4). The affinities of the resulting SMDCs **21–1** and **21–3** for integrin α_V_β_3_ were assessed using an ELISA‐like assay (Table 1). As expected, the stereochemically modified SMDC **21–3** exhibited a 34‐fold lower affinity than SMDC **21‐1**, consistent with the behavior of unconjugated mimetics. These results indicate that conjugation does not compromise integrin binding.

**SCHEME 4:**
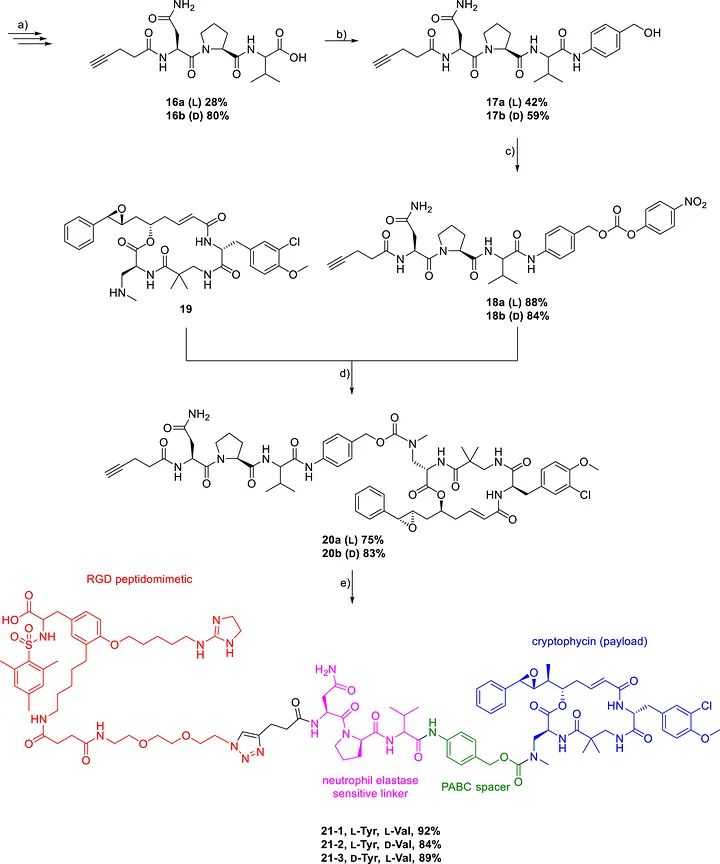
Synthesis of RGD peptidomimetic cryptophycin conjugates **21–1** (positive control), **21–2** (non‐cleavable), **21–3** (negative control). (a) SPPS starting from Fmoc‐l/d‐Val‐OH; (b) PAB‐OH, EEDQ, DCM/MeOH (2:1), RT, 24 h; (c) Bis‐PNP‐carbonate, DIPEA, DMF, RT, 18 h; (d) DIPEA, DMF, RT, 18 h; (e) **15a/b**, CuPF_6_(ACN)_4_, THPTA, DMF/H_2_O (3:2), RT, 1 h. a = l‐configured and b = d‑configured.

### 2.3 Biological Activity of SMDCs

For efficient delivery of the cryptophycin payload **19** to tumor tissue overexpressing integrin α_V_β_3_, efficient cleavage by neutrophil elastase and high stability of the SMDC in circulation are essential. All synthesized SMDCs were highly stable (>90 % of parent drug remaining, measured by LC–MS) in human plasma at 37°C for over 24 h, as shown in Figure S3. Cleavage behavior was determined by using human neutrophil elastase in a molar substrate‐to‐enzyme ratio of 1000:1. SMDC **21–1** showed rapid cleavage (<20 % of parent drug remaining) by human neutrophil elastase after 15 min, with maximum payload release observed after 2 h (Figure S4). In contrast, its d‐Val linker epimer **21–2** showed no release of the payload after 4 h, confirming non‑cleavability (Figure S5). The weakly binding d‐Tyr SMDC **21–3** displayed slightly reduced cleavability (<25 % of parent drug remaining after 2 h) compared to **21–1**. We further evaluated the in vitro cytotoxicity of SMDC **21–1** and their epimers **21–2** and **21–3** in comparison to cryptophycin **19** itself in colorectal cancer cells (HT29) and melanoma cells (WM115 and M21‐L), both in the presence or absence of human neutrophil elastase and with or without a washout step after 2 h (Table S3). In experiments including a washout step with neutrophil elastase present, the enzyme was added together with fresh culture medium after the washout. SMDC **21‐1** exhibited high cytotoxic activity against all cancer cell lines, with IC_50_ values in the single‐digit nanomolar range. Upon addition of neutrophil elastase to the culture medium, potency increased to reach levels similar to cryptophycin **19,** with IC_50_ values in the double‐digit picomolar range (Table 2). An additional washout step increased the IC_50_ values approximately twofold, suggesting that cellular uptake or cleavage is mostly completed within 2 h. In comparison, the non‐cleavable epimer **21–2** exhibited approximately 20–50‐fold lower cytotoxic activity across all cell lines. Contrary to the results obtained from the cleavage assay, addition of neutrophil elastase led to a 20–30‐fold increase in cytotoxic activity; however, this behavior is also described in the literature [28]. Surprisingly, comparison of IC_50_ values for SMDC **21–1** in integrin‑positive WM115 (α_v_+, α_v_β_3_+, α_5_β_1_+, α_v_β_6_‐, α_v_β_8_+) and integrin‐negative M21‐L cells (α_v_‐, α_v_β_3_‐, α_5_β_1_+, α_v_β_6_‐, α_v_β_8_‐), determined by flow cytometry [40], revealed insufficient selectivity between the two. Even though RGD mimetic **15a** binds highly selective to isolated integrin α_v_β_3_ compared to α_5_β_1_, making the M21‐L cells suitable as negative control [40], these results contradict previously described SMDCs, which showed a targeting index of 134 based on these cell lines [27]. Furthermore, SMDC **21–1** and its weakly binding epimer **21–3** exhibited similar IC_50_ values across all assays, even without any elastase present. A similar observation was reported by Lerchen et al. [28]. These findings may be attributed to the presence of endogenous elastase or unspecific uptake processes, such as hydrophobic interactions, which could potentially be mitigated by using a hydrophilicity‐inducing linker, a strategy currently under investigation [41]. Moreover, the effect of integrin binding would not influence the outcome of an in vitro cytotoxicity assay upon addition of elastase. In addition, the use of a highly potent drug, such as cryptophycin, may mask selectivity because even small amounts of internalized compound are sufficient to induce cell death. We further investigated the behavior of SMDC **21–1** in a temperature‐controlled cytotoxicity assay by incubating the compound at 4°C prior to a washout, a condition under which an energy‐dependent uptake like receptor‐mediated endocytosis is expected not to occur [42, 43]. However, no difference in IC_50_ values were observed between the two conditions (Figure S7). This result supports the previous assumption that non‐receptor‐mediated pathways contribute to cellular uptake. Additionally, SMDC **21–1** proved stable over 72 h in culture medium containing 10% fetal bovine serum, excluding the possibility of premature release of the cryptophycin during the in vitro assay (Figure S8).

**TABLE 2:** IC_50_ values (nM) of SMDCs in comparison to payload **19** in presence or absence of human neutrophil elastase (hNE) in HT29 and WM115 cancer cell lines. IC_50_ values were obtained by sigmoidal dose response fitting and were calculated as the mean values of at least three independent measurements.

Compound	HT29	HT29 + hNE	WM115	WM115 + hNE
**19**	0.02	0.01	0.03	0.02
**21–1**	5.20	0.07	6.7	0.14
**21–2**	114	1.97	109	3.80
**21–3**	5.46	0.05	7.8	0.11

## 3 Conclusion

A novel class of RGD peptidomimetic‐cryptophycin SMDCs employing a neutrophil elastase cleavable NPV‐PABC linker and a highly potent Dap(Me)‐derived cryptophycin payload have been synthesized. Optimization of the synthetic strategy for the Negishi cross‐coupling and Mitsunobu reactions enabled efficient and scalable access to stereochemically defined mimetics. The combination of mimetic and linker epimers in the corresponding SMDCs provided negative controls for both integrin binding and enzymatic cleavability. Biochemical studies showed rapid payload release by human neutrophil elastase with maximum release after 2 h, plasma stability remaining >90% over 24 h and preserved α_V_β_3_ integrin affinity (ELISA) upon conjugation. In vitro cytotoxicity assays confirmed that enzymatic cleavage restores cryptophycin activity to near free‐drug levels (IC_50_ (HT29): 0.02 nM), underscoring the effectiveness of the release mechanism. However, despite efficient cleavage and high potency, the SMDCs displayed limited integrin‐dependent selectivity, likely due to endogenous proteases or non‐specific uptake processes, as temperature‐controlled blocking of energy‐dependent uptake mechanisms still resulted in cytotoxic activity. In addition, the extreme potency of cryptophycin may mask receptor‐mediated differences. Efforts are ongoing to increase linker hydrophilicity and modulate physicochemical properties with the aim to reduce non‐specific uptake and improve tumor selectivity, thereby advancing the rational development of cryptophycin‐based targeted therapeutics.

## Conflicts of Interest

The authors declare no conflicts of interest.

## Supporting information



The authors have cited additional references within the Supporting Information [44].
**Supporting File 1**: chem71270‐sup‐0001‐SuppMat.docx

## Data Availability

The data that support the findings of this study are available in the supplementary material of this article.
