# Supplementary material for: Extracellularly Activatable Conjugates of RGD Peptidomimetics and Cryptophycin for αVβ3‐Targeted Cancer Therapy
Source: Chemistry. 2026 Jun 18;32(37):e71270. doi: 10.1002/chem.71270 (PMC13628569; doi:10.1002/chem.71270)
Supplement: Supplementary file 1 [file CHEM-32-e71270-s001.docx]

**Extracellularly Activatable Conjugates of RGD Peptidomimetics and Cryptophycin for α_v_β_3_-Targeted Cancer Therapy**

**Supporting Information**

Table of Contents

[1	Experimental Section	4](#_Toc230270625)

[1.1	General	4](#_Toc230270626)

[1.2	Biological Data	8](#_Toc230270627)

[1.2.1	ELISA-Like Assay	8](#_Toc230270628)

[1.2.2	Plasma Stability Assay	11](#_Toc230270629)

[1.2.3	Cleavage Assay	12](#_Toc230270630)

[1.2.4	Cytotoxicity Assay	16](#_Toc230270631)

[1.2.5	Culture Medium Stability Assay	21](#_Toc230270632)

[1.3	Synthetic Procedures	22](#_Toc230270633)

[1.3.1	General Procedures	22](#_Toc230270634)

[1.3.2	RGD-mimetic	23](#_Toc230270635)

[1.3.3	Linker	43](#_Toc230270636)

[1.3.4	Linker-Crypthophycins	50](#_Toc230270637)

[1.3.5	SMDCs	52](#_Toc230270638)

[2	Literature	54](#_Toc230270639)

[3	Appendix	55](#_Toc230270640)

[3.1	List of abbreviations	55](#_Toc230270641)

[3.2	Spectra	57](#_Toc230270642)

# 1 Experimental Section

## 1.1 General

**Working Techniques**

Air and water sensitive reactions were performed under Ar-atmosphere (Linde, quality grade 4.6, purity 99.996 %) using Schlenk-technique and dried out glassware. Removing solvents under reduced pressure was done using a rotary evaporator at 40 °C. Residual solvent was removed in vacuum (10‑^2^ mbar) or by freeze-drying (10^-1^ mbar). The temperature given for the reactions refers to the bath temperature. Unless otherwise stated, degassed solutions were obtained through three consecutive cycles of *freeze-pump-thaw*. Rotamers and their molar ratio were determined by high temperature (20-80 °C, in 20 °C steps) ^1^H-NMR-spectroscopy. The composition of solvent mixtures is given in volumetric ratios. Analyses by TLC were carried out on silica gel plates (Merck, F254) using UV-light (254 nm) or appropiate stains for visualization.

**Chemicals and Solvents**

All chemicals were purchased commercially in appropiate quality and, unless otherwise stated, were used without further purification. The solvents used for extraction and chromatography were of technical grade and distilled prior to use. DMF and solutions thereof were stored and handled under eclusion of light. Dry solvents have been absolute as follows:

DCM:	pre-dried over CaCl_2_, distilled over CaH_2_

THF:	pre-dried over KOH and CaCl_2_, distilled over Na / benzophenone

DMF:	dried over 4 Å molceular sieve

**Manual Column Chromatography**

Column chromatography was carried out with silica gel (Merck, Silica 60M, 0.040 – 0.063 mm) using eluents as specified. The size of the column is given as diameter x length. Fractions obtained by column chromatography were combined by TLC analyses.

**Preparative MPLC**

Automatic column chromatography was done using a Büchi Revelris X2 or Büchi Pure C-815 Flash. Columns were used in quality and size recommended by manufacturer. For loading, the crude products were if possible dissolved and injected as liquid or were adsorbed on celite and injected using a solid loader. Detection was performed using a UV detector at multiple wavelengths (220 nm, 254 nm, 280 nm) and an ELSD sensor.

**Preperative RP-HPLC**

Preparative RP-HPLC was either performed on a Merck-Hitachi system (controller: D-7000, pump: L-7150, detector: L-7420) equipped with a C18(2)-column (Luna Phenomenex, 5 µm Silica, 100 Å, LC Column 250 x 10 mm) for small scale reactions or on a Shimadzu system (controller: SCL-40, pump: LC20-AP, detector: SPD-40V, fraction collector: FRC-10A) equipped with a C18(2)-column (Luna Phenomenex, 5 μm Silica, 100 Å, LC Column 21.2 mm × 250 mm, AXIA^TM^ Packed, Ea). A linear gradient with a flowrate of 10 mL∙min^-1^ at RT and UV-detection at λ = 220 nm were used. As mobile phase mixtures A (95% H2O, 4.9% MeCN, 0.1% of either TFA or formic acid) and B (95% MeCN, 4.9% H2O, 0.1% of either TFA or formic acid) were used. For purification of compounds containing Cryptophycin formic acid was used as additive for mobile phases A and B because of partly degredation of the epoxide function when using TFA as additive.

**Analytical RP-HPLC**

Analytical HPLC samples were recorded at a CBM-20A chromatography system (SHIMADZU) consisting of an autosampler (Nexera XR SIL-20Axr), pump (Nexera XR LC-20ADXR), column oven (CTO-20A), and diode array detector (SPD-M20A IVDD). As column a Phenomenex Luna C18(2) column (part number: 00D-4251-B0, pore size: 100 Å, particle size: 3 μm, length: 100 mm, internal diameter: 2.0 mm) at 40 °C column oven temperature was used. The mobile phase consisted of A (99.9% H2O, 0.1% TFA) and B (99.9% MeCN, 0.1% TFA) with the following percentages of B: 0 – 5.5 min, 5 – 95%; 5.5 – 6.0 min, 95%; 6.0 – 6.1 min, 95% – 5%; 6.1 – 9.0 min, 5% and had a flow rate of 0.60 mL/min. Usually, 5 μL of an aqueous solution of the analyte (~ 1 mM) was injected.

**Analytical HPLC-MS**

Analytical HPLC-MS measurements were recorded on one of the following devices:

**HPLC-MS:**

Agilent 1200 HPLC System (Agilent Technologies, Santa Clara, CA, USA) consisting of an autosampler, degasser, binary pump, column oven, and diode array detector. A Phenomenex Luna C18(2) column (part number: 00D-4251-B0, pore size: 100 Å, particle size: 3 μm, length: 100 mm, internal diameter: 2.0 mm) was used for separation and operated at 40 °C column oven temperature. The HPLC was directly coupled to an Agilent 6220 ESI-TOF mass spectrometer (Agilent Technologies, Santa Clara, CA, USA) using a Dual-ESI source at 2.5 kV spray voltage. Nitrogen generated by a nitrogen generator NGM 11 served both as the nebulizer gas and the dry gas. Spectra were recorded in a range of 150 to 2500 *m*/*z* with a rate of two spectra per second and analyzed with MassHunter Workstation Acquisition B.07.00 software. The mass spectrometer was calibrated with external standard (ESI-L Tuning Mix, Agilent Technologies, Santa Clara, CA, USA) as calibration standard. The mobile phase consisted of A (94.9% H2O, 5.0% MeCN, 0.1% formic acid) and B (94.9% MeCN, 5.0% H2O, 0.1% formic acid) with the following percentages of B: 0 – 10 min, 0 – 98%; 10 – 11 min, 98%; 11 – 11.5 min, 98% – 0%; 11.5 – 15 min, 0% and had a flow rate of 0.3 mL/min. Usually, 5 μL of an aqueous solution of the analyte (~ 1 mM) was injected.

**UHPLC-MS:**

UltiMate 3000RS UHPLC System (Thermo Fisher Scientific) consisting of an autosampler, degasser, pump, column oven and diode array detector. The UHPLC was directly coupled to an AmaZonX ESIion trap mass spectrometer (Bruker) using an ESI source at 4.5 kV spray voltage. Nitrogen generated by a nitrogen generator G6-0-1-0-E (Parker-Hannifin, model G4600E) served both as the nebulizer S10 gas and the dry gas. Spectra were recorded in a range of 100 to 2800 *m*/*z* with a rate of five spectra per second and analyzed with Compass DataAnalysis 4.2 software. The mass spectrometer was calibrated with external Standard (ESI-T Tuning Mix, Agilent Technologies, Santa Clara, CA, USA) as calibration standard. A Phenomenex Kinetex C18 column (part number: 00D-4475-AN, pore size: 100 Å, particle size: 1.7 μm, length: 100 mm, internal diameter: 2.1 mm) was used for separation. The column was operated at 60 °C oven temperature. The mobile phase consisted of A (94.9% H2O, 5.0% MeCN, 0.1% formic acid) and B (94.9% MeCN, 5.0% H2O, 0.1% formic acid) with the following percentages of B: 0 – 3.5 min, 0 – 100%; 3.5 – 4 min, 100%; 4 – 4.5 min, 100% – 0%; 4.5 – 5 min, 0% and had a flow rate of 0.75 mL/min. Usually, 5 μL of an aqueous solution of the analyte (~ 1 mM) was injected.

**NMR Spectroscopy**

NMR spectra were recorded on a Bruker Avance III 500, Bruker Avance III 500 HD (500.20 MHz for ^1^H, 125.79 MHz for ^13^C) or Bruker Avance III 600 (600.13 MHz for ^1^H, 150.90 MHz for ^13^C) at 298 K. The chemical shift (δ) is given in ppm realtive to TMS (δ_TMS_ = 0 ppm) and is referenced to the residual hydrogen signal of the solvent as follows:

C_2_*H*D_5_OS	δ 2.50 ppm (^1^H-NMR) and δ 39.5 ppm (^13^C-NMR)
C*H*Cl_3_	δ 7.26 ppm (^1^H-NMR) and δ 77.2 ppm (^13^C-NMR)
C*H*D_2_OD	δ 3.31 ppm (^1^H-NMR) and δ 49.0 ppm (^13^C-NMR)

Signal assignment of NMR spectra (^1^H, ^13^C{^1^H}) was supported by 1D- and 2D-NMR spectra (^1^H,^1^H-COSY, ^1^H,^1^H-TOCSY, ^13^C-DEPT-135, ^1^H,^13^C-HMQC, ^1^H,^13^C-HMBC). The yields of products containing solvent residues, by-products or impurities that could be structurally identified by ^1^H NMR spectroscopy were corrected through integration of the ^1^H NMR spectra.

**Mass Spectrometry**

*These protocols were adopted from and with permission of the mass department University Bielefeld:* Nano-ESI mass spectra were recorded using an Esquire 3000 ion trap mass spectrometer (Bruker Daltonik GmbH, Bremen, Germany) equipped with a nano-ESI source. Samples were dissolved in a suitable solvent (usually acetonitrile) and introduced by static nano-ESI using in-house pulled glass emitters. Nitrogen served both as nebulizer gas and dry gas. Nitrogen was generated by a Bruker nitrogen generator NGM 11. Helium served as cooling gas for the ion trap. The mass axis was externally calibrated with ESI-L Low Concentration Tuning Mix (Agilent Technologies, Santa Clara, CA, USA) as calibration standard.

Synapt G2Si nano-ESI measurements are performed using a Q-IMS-TOF mass spectrometer Synapt G2Si (Waters GmbH, Manchester, UK) in resolution mode, interfaced to a nano-ESI. Nitrogen serves both as nebulizer and dry gas for nano-ESI. Samples were dissolved in acetonitrile and introduced by static nano-ESI using in-house pulled glass emitters. The mass axis was internally calibrated with the protonated LeuEnk ion as internal calibration standard. Scan accumulation and data processing was performed with MassLynx 4.1 (Waters GmbH, Manchester, UK) on a PC Workstation. The spectra shown were generated by the accumulation and averaging of several single spectra. Determination of exact masses were performed using centroided data.

## 1.2 Biological Data

### 1.2.1 ELISA-Like Assay

IC_50_ values were determined using the extracellular domains of the integrins in accordance to the literature.^[1]^

**Preparation of proteins**

Commercially available Vitronectin (100 µg, R&D Systems, No.: 2349-VN-100) was reconstituted in PBS buffer at 100 µg∙mL^-1^ according to manufacturer protocol, and was dispensed into 50 µL aliquots, which were stored at -80 °C. Recombinant Human Integrin α_v_β_3_ (50 µg, R&D Systems, No.: 3050-AV-050) was reconstituted in PBS-T buffer at 100 µg∙mL^-1^ and was dispensed into 50 µL aliquots, which were stored at -80 °C until usage. Mouse Anti-Human CD51/61 (100 µg, 0.5 mg∙mL^‑1^, BD Biociences, No.: 555504) was stored undiluted at 4 °C. Anti-Mouse-IgG POD goat (1 mL, 0.76 mg∙mL^-1^, Sigma‑Aldrich, No.: A4416-1ML) was dispensed into 26.4 µL aliquots and stored at -80 °C until usage.

**Table S1:** Buffers applied in the ELISA-like Assay.

| Buffer | Composition |
| --- | --- |
| Carbonate | 15.0 mM Na_2_CO_3_, 35.0 mM NaHCO_3_, pH 9.6 |
| PBS-T | 137 mM NaCl, 2.70 mM KCl, 10.0 mM Na_2_HPO_4_, 0.01 % Tween20, pH 7.4 |
| TS-B | 20.0 mM Tris-HCl, 150 mM NaCl, 1.00 mM MgCl_2_, 1.00 mM MnCl_2_, 1 % BSA, pH 7.5 |

**Protocol**

Vitronectin (100 µL, 100 µg∙mL^-1^) was diluted with carbonate buffer (10.5 mL). 100 µL of this solution (1.00 µg∙mL^-1^) was added to each well of a flat-bottom 96-well immune plate (Brand) and incubated at 6 °C over night. Alle wells were washed with PBS-T (3x200 µL/well) and blocked with TS-B (150 µL/well) for 1 h at RT. In the meantime a second plate with a dilution series of the internal standard (Cilengitide, 2.00 mM DMSO stock solution diluted to 0.80 µM with TS-B, then 1:4 dilution with TS-B from wells B-G) and compounds (RGD-mimetic or SMDCs, 2.00 mM DMSO stock solution diluted to 50.0 µM with TS-B, then 1:9 dilution with TS-B from wells B-G) were prepared. Furthermore, Recombinant Human Integrin α_v_β_3_ (100 µL, 100 µg∙mL^-1^) was diluted with TS-B (5.25 mL). Afterwards all wells of the main plate were washed with PBS-T (3x200 µL/well) as well as the buffer thoroughly removed and 50 µL of the dilution series were added to wells B‑G (two technical replicates for the internal standard, five technical replicates for compounds). To wells A (negative control) 100 µL/well and to wells H (positive control) 50 µL/well of TS-B were added. Afterwards the solution of integrin α_v_β_3_ in TS-B (2.00 µg∙mL^-1^, 50 µL/well) were added to wells B-G and the plate incubated for 1 h at RT. After washing the assay plate with PBS-T (3x200 µL/well) a solution of Mouse Anti-Human CD51/61 (2.00 µg∙mL^-1^ in TS-B, 100 µL/well) was added to each well and incubated for 1 h at RT. Washing and incubation of Anti-Mouse-IgG POD goat (1.00 µg∙mL^-1^ in TS-B, 100 µL/well) were done as described above. Afterwards the plate was washed with PBS-T (3x200 µL/well) and treated with SeramunBlau fast2 (Seramun Diagnostics GmbH, 50µL/well) under exclusion of light. The staining was stopped by addition of H_2_SO_4_ (3 M, aq., 50 µL/well) a blue color gradient from well A to H could be observed (~2 min). The absorbance was measured with a plate reader at 450 nm and corrected by subtraction of the absorbance at 620 nm. Using OriginPro 2022 the resulting values were plotted and analyzed. Each data point of compounds refers to the mean value of at least 3 technical replicates. The inflection point of a resulting DoseResp fit describes the IC_50_ value.

**Figure S1:** Inhibition of the binding of immobilized vitronectin to isolated integrin α_v_β_3_ by RGD-mimetics **15a-b** in comparison to cilengitide. IC_50_(Cilengitide) = 6.34 ± 1.32 nM, IC_50_(**15a**) = 150 ± 24.1 nM, IC_50_(**15b**) = 4745 ± 265 nM. Data points are presented as mean values with standard deviations from at least 3 technical replicates. IC_50_ values were obtained by sigmoidal dose response fitting, and error values represent the standard error of the fitted parameter.

 **Figure S2:** Inhibition of the binding of immobilized vitronectin to isolated integrin α_v_β_3_ by RGD-mimetics **21-1** and **21-3** in comparison to cilengitide. IC_50_(Cilengitide) = 6.34 ± 1.32 nM, IC_50_(**21-1**) = 62.6 ± 4.27 nM, IC_50_(**21-3**) = 2126 ± 254 nM. Data points are presented as mean values with standard deviations from at least 3 technical replicates. IC_50_ values were obtained by sigmoidal dose response fitting, and error values represent the standard error of the fitted parameter.

### 1.2.2 Plasma Stability Assay

For stability measurements leukocyte-depleted fresh frozen plasma (with CPD) provided by the Herz- und Diabeteszentrum NRW (0.75 to 0.82 mL∙mL^-1^ coagulation-active human plasma from a single whole blood donation, CPD stabilizer (Ph.Eur.), residual cell content: erythrocytes < 6.0 x 109/L, leukocytes < 1 x 106/unit, thrombocytes < 50 x 109/L) was used.

**Protocol**

Above-described human plasma was preincubated at 37 °C for 15 minutes. For t = 0 min stopping solution (same volume as reaction mixture, 0.2 % formic acid and 32 µM Warfarin in ACN) was added and then a solution of corresponding compound (20 µL in a mixture of ACN/DMSO (1:1, v:v)) added to each vial to give a 60 µM suspension regarding corresponding compound. The reaction was stopped at t = 0, 15, 30, 45, 60, 120, 240 and 1440 min by addition of stopping solution (same volume as reaction mixture) and cooling to 0 °C. Samples were centrifuged and analyzed by UHPLC-MS using DataAnalysis 4.2. Warfarin was used as internal standard. Each data point of compounds corresponds to the mean value of the UV signal (220 nm) of 3 technical replicates.

 **Figure S3:** Plot of the relative concentration of SMDC **21-1-3** in presence of leukocyte-depleted fresh frozen human citrate plasma against the reaction time t. Reaction mixture containing 60 µM SMDC and 2-3 % DMSO. Data points are presented as mean values with standard deviations from 3 technical replicates.

### 1.2.3 Cleavage Assay

Protocols were modified and adopted from Lerchen.^[2]^

**Preparation of Enzyme**

Commercially available human neutrophil elastase (50µg, Sigma-Aldrich, No.: 324681-50UG) reconstituted in aqueous buffer (50 mM sodium acetate, 200 mM NaCl, pH 5.5) at 50 µg∙833 µL^-1^ (2 µM) was according to manufacturer protocol, and was dispensed into 100 µL aliquots, which were stored at ‑20 °C.

**Table S2:** Buffers and solutions applied in the cleavage assay.

| Buffer | Composition |
| --- | --- |
| Assay buffer | 100 mM Tris, 150 mM NaCl, 10 mM CaCl_2_, pH 7.5 |
| Stopping solution | 0.2 % formic acid and 32 µM Warfarin in ACN |

**Protocol**

Assay buffer (75 µL for negative control and 50 µL for cleavage assay) was preincubated in PCR-stripes (Sarstedt AG & Co. KG, Multiply-Pro cup 0.2 mL, PP) at 37 °C for 30 minutes. A solution of the corresponding compound (25µL, 235 µM in assay buffer containing 30% DMSO) was added to each vial. For t = 0 h stopping solution (100 µL) was added and the reaction was started by addition of the enzyme (25 µL, 240 nM in assay buffer) to each vial. The reaction was stopped at 0, 15, 30, 45, 60, 120, 240 minutes by addition of stopping solution (100 µL) and cooling to 0 °C. Samples were centrifuged and analyzed by UHPLC-MS using DataAnalysis 4.2. Warfarin was used as internal standard. Each data point of compounds corresponds to the mean value of the UV signal (220 nm) of 3 technical replicates.

 **Figure S4:** Plot of the relative concentration of SMDC 21-1 and the released drug against the reaction time t in presence or absence of human neutrophil elastase (hNE) with a molar substrate to enzyme ratio of 1000:1. Measured data are reported with their standard deviations. Data points are presented as mean values with standard deviations from 3 technical replicates.

 **Figure S5:** Plot of the relative concentration of SMDC **21-2** and the released drug against the reaction time t in presence or absence of human neutrophil elastase (hNE) with a molar substrate to enzyme ratio of 1000:1. Measured data are reported with their standard deviations. Data points are presented as mean values with standard deviations from 3 technical replicates.

 **Figure S6:** Plot of the relative concentration of SMDC **21-3** and the released drug against the reaction time t in presence or absence of human neutrophil elastase (hNE) with a molar substrate to enzyme ratio of 1000:1. Measured data are reported with their standard deviations. Data points are presented as mean values with standard deviations from 3 technical replicates.

### 1.2.4 Cytotoxicity Assay

The HT29 (RRID: CVCL_0320) and WM115 (RRID: CVCL_0040) cells were cultivated in Dulbecco’s modified Eagle medium (DMEM, PAN Biotech, Cat. No. P04-03590) with glucose (4.5 g∙L^-1^), sodium bicarbonate (3.7 g∙L^-1^), sodium pyruvate, l-glutamine and phenol red, supplemented with 10 % fetal bovine serum (FBS Supreme, South America origin, 0.2 µM sterile filtered, PAN Biotech, Cat. No. P30-3031) and 1 % penicillin-streptomycin (1x, PAN Biotech, Cat. No. P06-07100). 1 % NEAA (1x, Pan Biotech, Cat. No. P08-32100) is added for the WM115 cells. M21-L (RRID: CVCL_JZ12) cells were cultivated in Roswell Park Memorial Institute 1640 medium (RPMI, PAN Biotech, Cat. No. P04-16500) with sodium bicarbonate (2.0 g∙L^-1^), l-glutamine, glucose and phenol red, supplemented with 10 % fetal bovine serum (FBS Supreme, South America origin, 0.2 µM sterile filtered, PAN Biotech, Cat. No. P30-3031) and 1 % penicillin-streptomycin (1x, PAN Biotech, Cat. No. P06-07100). The cells were maintained at 37 °C and 5.3 % CO_2_/humidified air. On the day before the test, the cells (80 % confluence for adherent cells) were detached with trypsin/ethylenediaminetetraacetic acid (EDTA) solution (0.05 %/0.02 % in Dulbeco’s Phosphate Buffered Saline [DPBS]) and plated in sterile 96-well plates in a density of 3000 cells in 50 µL medium per well. The dilution series of the compounds were prepared in an external sterile 96-well plate from stock solutions in DMSO of concentrations of 2 mM to 5 mM. Some culture medium was added to the wells to adjust the volume of the wells to the wanted dilution factor and dilution was done pipetting liquid from well to well. Final concentrations were achieved by addition of 50 µL of the dilution series to 50 µL of culture medium. In case of the assays with neutrophil elastase, 40 µL of the dilution series and 10 µL of the enzyme in aqueous buffer (50 mM sodium acetate, 200 mM NaCl, pH 5.5) was used to achieve a final concentration of the enzyme of 20 nM. In case of the assays with a washout, the supernatant was removed after 2 h and replaced with 100 µL of culture medium. In case of the assays with a washout and neutrophil elastase present, the supernatant was removed after 2 h and replaced with 90 µL of culture medium and 10 µL of the enzyme in aqueous buffer (50 mM sodium acetate, 200 mM NaCl, pH 5.5). After incubation for 72 h at 37 °C and 5.3% CO_2_/humidified air, 30 μL of a 175 μM aqueous resazurin solution (116 mM NaCl, 26 mM NaHCO_3_, pH = 7.3) was added to each well. The cells were incubated at the same conditions for 6 h. Subsequently, fluorescence was measured. The excitation was effected at a wavelength of 530 nm, whereas the emission was recorded at a wavelength of 588 nm. The IC_50_ values were calculated as a sigmoidal dose response curve using GraphPad Prism 4.03. Concentrations were measured in duplicates and replicated at least three times. IC_50_ values (Table S3) were calculated as the mean value of each independent measurement.

**Table S3:** IC_50_-Values (nM) of SMDCs in comparison to the payload in presence or absence of 20 nM hNE in WM115, HT29 and M21-L cancer cell lines with or without washout after 2 h incubation. IC_50_ values were obtained by sigmoidal dose response fitting and were calculated as the mean value of at least 3 independent measurements.

| SMDCs | HT29 | HT29  + Enzyme | HT29  +   wash-out | HT29  + Enzyme wash-out | WM115 | WM115  + Enzyme | WM115  +   wash-out | WM115  + Enzyme wash-out | M21-L | M21-L  + Enzyme | M21-L  +   wash-out | M21-L  + Enzyme wash-out |
| --- | --- | --- | --- | --- | --- | --- | --- | --- | --- | --- | --- | --- |
| Payload | 0.02 | 0.01 | 0.17 | 0.22 | 0.03 | 0.02 | 0.25 | 0.29 | 0.12 | 0.11 | 1.2 | 0.85 |
| **21-2** | 114 | 1.97 | 499 | 14.9 | 109 | 3.8 | 363 | 15 | 266 | 13 | 669 | 34 |
| **21-1** | 5.20 | 0.07 | 9.1 | 0.26 | 6.7 | 0.14 | 12 | 0.21 | 9.6 | 0.31 | 14.3 | 0.39 |
| **21-3** | 5.46 | 0.05 | 9.7 | 0.31 | 7.8 | 0.11 | 21 | 0.44 | 12 | 0.31 | 25.9 | 0.69 |

#### 1.2.4.1 Temperature controlled Cytotoxicity Assay

Unlike the protocol described above, the cells and dilution series were cooled to 6 °C for 20 min prior to incubation for 2 h at the same temperature. The subsequent washout step was performed on a cooling pack to maintain the temperature. All following steps were carried out as described in the standard protocol.

**Figure S7**: Comparison of IC_50_-Values of SMDC **21-1** obtained in a cytotoxicity assay by standard protocol (warm) or temperature controlled (cold). Data points are presented as mean values with standard deviations from at least 3 technical replicates. IC_50_ values were obtained by sigmoidal dose response fitting.

### 1.2.5 Culture Medium Stability Assay

Dulbecco’s modified Eagle medium (DMEM, PAN Biotech, Cat. No. P04-03590) with glucose (4.5 g∙L^-1^), sodium bicarbonate (3.7 g∙L^-1^), sodium pyruvate, l-glutamine, phenol red and 1 % penicillin-streptomycin (1x, PAN Biotech, Cat. No. P06-07100) was supplemented either with 10 % fetal bovine serum (FBS Supreme, South America origin, 0.2 µM sterile filtered, PAN Biotech, Cat. No. P30-3031) and protease inhibitor cocktail set I (1x, Calbiochem, Cat. No. 539131), with 10 % FBS only, or left unchanged. The different culture media (90 µL) were preincubated in PCR-stripes (Sarstedt AG & Co. KG, Multiply-Pro cup 0.2 mL, PP) at 37 °C for 10 minutes. For t = 0 h stopping solution (100 µL, acetonitrile containing 0.2 % formic acid and 0.6 mg/mL biphenyl) was added and the reaction was started by addition of a solution of conjugate **21-1** (10 µL, 600 µM in DMSO and water (1:1, v:v)) to each vial. The reaction was stopped at 0, 24, 48, 72 hours by addition of stopping solution (100 µL) and cooling to 0 °C. Samples were centrifuged and analyzed by UHPLC-MS using DataAnalysis 4.2. Biphenyl was used as internal standard. Each data point of compounds corresponds to the mean value of the UV signal (220 nm) of 3 technical replicates.

**Figure S8:** Plot of the relative concentration of SMDC **21-1** in presence of DMEM supplemented either with 10 % FBS and protease inhibitor cocktail set I (PCIS), with 10 % FBS only, or left unchanged against the reaction time t. Reaction mixture containing 60 µM SMDC. Data points are presented as mean values with standard deviations from at least 3 technical replicates.

## 1.3 Synthetic Procedures

### 1.3.1 General Procedures

#### 1.3.1.1 GP I: Determination of Resin Loading

The dry and loaded resin (~1.00 mg) was added to a UV quartz cuvette and incubated with cleavage solution (3 mL, 20 % piperidine in DMF) for 1 h at RT. Afterwards the absorption of the solution (reference: 20 % piperidine in DMF) at 290 nm was measured using a UV-Vis spectrometer. The loading can be determined using following equation:

$$\mathrm{loading}\left[ \frac{\text{mmol}}{\text{g}} \right]\text{=}\frac{\text{A}_{\text{290nm}}}{\text{m}_{\text{resin[mg]}}\text{∙1.65}}$$

(1)

#### 1.3.1.2 GP II: Fmoc Cleavage

Cleavage solution (20 % piperidine in DMF, + 0.1 % HOBt) was added to the resin and the syringe was shaken for 30 minutes at RT. Afterwards the suspension was filtered and the resin washed with DMF (3x10 mL). This procedure was applied twice to ensure complete deprotection.

#### 1.3.1.3 GP III: Peptide Coupling

A solution of Fmoc-L/D-AA(PG)-OH or 4-Pentynoic acid (4 eq.), Oxyma (4 eq.) and DIC (4 eq.) in DMF (12 mL) was added to the resin and the syringe was shaken for 3 h at RT. The suspension was filtered and the resin washed with DMF (3x10 mL).

#### 1.3.1.4 GP IV: Test Cleavage

A small amount of dried resin (~10 beads) was suspended in a mixture of TFA/TIS/H_2_O (95:2.5:2.5, v:v:v) and incubated for 30 minutes at RT. Afterwards the sample was concentrated in vacuum and the crude suspended in a mixture of ACN/H_2_O (1:1, v:v, 200 µL). After centrifugation the supernatant was analyzed by LCMS.

### 1.3.2 RGD-mimetic

#### 1.3.2.1 (*S*)-2-amino-3-(4-hydroxy-3-iodophenyl)propanoic acid (2a)

l-Tyrosine (10.0 g, 55.2 mmol, 1.0 eq.) was dissolved in ammonia (500 mL, 25%, aq.). Iodine (14.0 g, 55.2 mmol, 1.0 eq.) was dissolved in ethanol (250 mL) and added dropwise over 1 h, while cooling to -10 °C. Afterwards the solvent was removed under reduced pressure and the crude suspended in ice cold acetone (250 mL). Büchner filtration, additional washing with ice cold acetone (200 mL) and drying under HV yielded a slight yellow solid (12.9 g) containing a iodo-l-tyrosine **2a** (11.2 g, 36.6 mmol, 66 %) in a mixture with diiodo-l-tyrosine (639 mg, 1.47 mmol, 3 %), l‑tyrosine (895 mg, 4.94 mmol, 9 %) and ­­acetone (108 mg, 1.86 mmol). This mixture was used in the next step without further purification.

M: 307.09 g/mol | Yield: 66 %, 11.2 g, 41.9 mmol | C_9_H_10_INO_3_

^1^**H-NMR** (500 MHz, methanol-*d*_4_):

δ [ppm] = 7.65 (d, ^4^*J* = 2.3 Hz, 1H, C^2^-**H**), 7.14 (dd, ^3^*J* = 8.3 Hz, ^4^*J* = 2.1 Hz, 1H, C^6^-**H**), 6.81 (d, ^3^*J* = 8.2 Hz, 1H, C^5^-**H**), 3.70 (dd, ^3^*J* = 8.6 Hz, ^3^*J* = 4.4 Hz, 1H, C^α^-**H**), 3.17 (dd, ^2^*J* = 14.7 Hz, ^3^*J* = 4.5 Hz, 1H, C^β^-**H**/H’), 2.90 (dd, ^2^*J* = 14.7 Hz, ^3^*J* = 8.4 Hz, 1H, C^β^-H/**H’**).

#### 1.3.2.2 (*R*)-2-amino-3-(4-hydroxy-3-iodophenyl)propanoic acid (2b)

d-Tyrosine (10.0 g, 55.2 mmol, 1.0 eq.) was dissolved in ammonia (500 mL, 25%, aq.). Iodine (14.0 g, 55.2 mmol, 1.0 eq.) was dissolved in ethanol (250 mL) and added dropwise over 1 h, while cooling to -10 °C. Afterwards the solvent was removed under reduced pressure and the crude suspended in ice cold acetone (250 mL). Büchner filtration, additional washing with ice cold acetone (200 mL) and drying under HV, as well as refiltration of the mother liquor yielded a slight yellow solid (18.9 g) containing a iodo-d-tyrosine **2b** (15.6 g, 50.7 mmol, 92 %) in a mixture with diiodo-d-tyrosine (2.42 g, 5.58 mmol, 10 %) and d‑tyrosine (874 mg, 4.82 mmol, 9 %). This mixture was used in the next step without further purification.

M: 307.09 g/mol | Yield: 92 %, 15.6 g, 50.8 mmol | C_9_H_10_INO_3_

^1^**H-NMR** (500 MHz, methanol-*d*_4_):

δ [ppm] = 7.65 (s, 1H, C^2^-**H**), 7.14 (d, ^3^*J* = 8.3 Hz, 1H, C^6^-**H**), 6.81 (d, ^3^*J* = 8.2 Hz, 1H, C^5^-**H**), 3.70 (dd, ^3^*J* = 7.8 Hz, ^3^*J* = 4.1 Hz, 1H, C^α^-**H**), 3.18 (dd, ^2^*J* = 14.6 Hz, ^3^*J* = 3.5 Hz, 1H, C^β^-**H**/H’), 2.90 (dd, ^2^*J* = 14.8 Hz, ^3^*J* = 8.6 Hz, 1H, C^β^-H/**H’**).

#### 1.3.2.3 Methyl (*S*)-2-((tert-butoxycarbonyl)amino)-3-(4-hydroxy-3-iodophenyl)propanoate (3a)

A mixture (14.8 g) of methyl ester **S1a**, diiodo-l‑tyrosine methyl ester and l-tyrosine methyl ester in a molar ratio of 90:2:8 and Boc_2_O (13.7 g, 62.7 mmol, 1.7 eq. regarding **2a**) was dissolved in 100 mL of a mixture of THF/water (2:1, v:v). The pH-value was adjusted to pH=10 by addition of NaOH (1 M, aq.) and the resulting suspension stirred overnight at RT. After acidification with HCl (1 M, aq.) to pH=2 the phases got separated and the aqueous phase was extracted with ethyl acetate (2x100 mL). The combined organic layers were washed with brine and dried over MgSO_4_. After removing the solvent under reduced pressure, purification by automatic MPLC and freeze-drying Boc-protected methyl ester **3a** (7.65 g, 18.2 mmol, 50 % over 2 steps) was obtained.

M: 421.23 g/mol | Yield: 50 % over 2 steps, 7.65 g, 18.2 mmol | C_15_H_20_INO_5_

**DC**: R*_f_* (cyclohexane/ethyl acetate, 2:1) = 0.26.

**UHPLC-MS** (ESI+):	*m*/*z* (experimental):	444.0966, t_R_ = 2.7 min.
*m*/*z* (experimental):	865.0661
*m*/*z* (calculated):	444.0278, [M+Na]^+^
*m*/*z* (calculated):	865.0664, [2M+Na]^+^

^1^**H-NMR** (500 MHz, methanol-*d*_4_):

δ [ppm] = 7.52 (d, ^4^*J* = 2.1 Hz, 1H, C^2^-**H**), 7.02 (dd, ^3^*J* = 8.3 Hz, ^4^*J* = 2.2 Hz, 1H, C^6^-**H**), 6.75 (d, ^3^*J* = 8.2 Hz, 1H, C^5^-**H**), 4.27 (dd, ^3^*J* = 9.0 Hz, ^3^*J* = 5.7 Hz, 1H, C^α^-**H**), 3.69 (s, 3H, -OC**H_3_**), 2.98 (dd, ^2^*J* = 13.9 Hz, ^3^*J* = 5.6 Hz, 1H, C^β^-**H**/H’), 2.77 (dd, ^2^*J* = 13.9 Hz, ^3^*J* = 9.0 Hz, 1H, C^β^-H/**H’**), 1.39 (s, 9H, Boc-**H**).

^13^**C**{^1^**H**}**-NMR** (126 MHz methanol-*d*_4_):

δ [ppm] = 174.1 (-**C**OOMe), 157.8 (^t^BuO**C**ONH-), 156.9 (Tyr-**C**^4^), 141.0 (Tyr-**C**^2^), 131.3 (Tyr-**C**^6^), 131.2 (Tyr-**C**^1^), 115.6 (Tyr-**C**^5^), 84.4 (Tyr-**C**^3^), 80.7 (-**C**(CH_3_)_3_), 56.6 (Tyr-**C**^α^), 52.6 (-**C**H_3_), 37.3 (Tyr‑**C**^β^), 28.7(-C(**C**H_3_)_3_).

#### 1.3.2.4 Methyl (*R*)-2-((tert-butoxycarbonyl)amino)-3-(4-hydroxy-3-iodophenyl)propanoate (3b)

A mixture (19.7 g) of methyl ester **S1b** (14.4 g, 44.8 mmol), diiodo-d-tyrosine methyl ester (1.65 g, 3.69 mmol), d-tyrosine methyl ester (0.96 g, 4.93 mmol) and Boc_2_O (20.3 g, 93.1 mmol, 2.0 eq.) was dissolved in 100 mL of a mixture of THF/water (2:1, v:v). The pH-value was adjusted to pH=10 by addition of NaOH (1 M, aq.) and the resulting suspension stirred overnight at RT. After acidification with HCl (1 M, aq.) to pH=2 the phases got separated and the aqueous phase was extracted with ethyl acetate (3x100 mL). The combined organic layers were washed with brine (1x100 mL) and dried over MgSO_4_. After removing the solvent under reduced pressure, purification by automatic MPLC and freeze-drying Boc-protected methyl ester **3b** (11.3 g, 26.9 mmol, 60 %) was obtained as colorless solid.

M: 421.23 g/mol | Yield: 60 %, 11.3 g, 26.9 mmol | C_15_H_20_INO_5_

**UHPLC-MS** (ESI+):	*m*/*z* (experimental):	843.0886, t_R_ = 2.7 min.
*m*/*z* (calculated):	843.0844, [2M+H]^+^

^1^**H-NMR** (500 MHz, methanol-*d*_4_):

δ [ppm] = 7.52 (s, 1H, C^2^-**H**), 7.02 (d, ^3^*J* = 8.3 Hz, 1H, C^6^-**H**), 6.75 (d, ^3^*J* = 8.2 Hz, 1H, C^5^-**H**), 4.27 (dd, ^3^*J* = 9.0 Hz, ^3^*J* = 5.6 Hz, 1H, C^α^-**H**), 3.69 (s, 3H, -OC**H_3_**), 2.98 (dd, ^2^*J* = 13.9 Hz, ^3^*J* = 5.5 Hz, 1H, C^β^-**H**/H’), 2.77 (dd, ^2^*J* = 13.9 Hz, ^3^*J* = 9.0 Hz, 1H, C^β^-H/**H’**), 1.39 (s, 9H, Boc-**H**).

^13^**C**{^1^**H**}**-NMR** (126 MHz, methanol-*d*_4_):

δ [ppm] = 174.1 (-**C**OOMe), 157.7 (^t^BuO**C**ONH-), 156.9 (Tyr-**C**^4^), 140.9 (Tyr-**C**^2^), 131.3 (Tyr-**C**^6^), 131.2 (Tyr-**C**^1^), 115.6 (Tyr-**C**^5^), 84.4 (Tyr-**C**^3^), 80.7 (-**C**(CH_3_)_3_), 56.6 (Tyr-**C**^α^), 52.6 (-**C**H_3_), 37.3 (Tyr‑**C**^β^), 28.7(-C(**C**H_3_)_3_).

#### 1.3.2.5 Benzyl (5-hydroxypentyl)carbamate (5)

5-Aminopentan-1-ol **(4)** (5.00 g, 48.4 mmol, 1.0 eq.) was dissolved in 100 mL of a mixture of acetone and water (1:1, v:v) and K_2_CO_3_ (10.0 g, 72.3 mmol, 1.5 eq.) was added. Cbz-Cl (15.0 g, 87.9 mmol, 1.8 eq.) was added portionwise while cooling with an ice bath. The reaction mixture was stirred overnight, slowly warming to RT. The resulting solution was then diluted with water and ethyl acetate and the phases separated. The aqueous phase was extracted with ethyl acetate (3x100 mL), and the combined organic phases were washed with HCl (1x100 mL, 1 M, aq.) and brine (1x100 mL) and dried over MgSO_4_. After removing the solvent under reduced pressure and purification via column chromatography (cyclohexane:ethyl acetate, 2:1), Cbz-protected alcohol **5** (7.79 g, 32.8 mmol, 68 %) was obtained as a colorless solid.

M: 237.30 g/mol | Yield: 68 %, 7.79 g, 32.8 mmol | C_13_H_19_NO_3_

**DC**: R*_f_* (cyclohexane/ethyl acetate, 1:1) = 0.1.

**HPLC-MS** (ESI+):	*m*/*z* (experimental):	260.1289, t_R_ = 7.0 min.
*m*/*z* (calculated):	260.1263, [M+Na]^+^

^1^**H-NMR** (500 MHz, CDCl_3_):

δ [ppm] = 7.38 – 7.28 (m, 5H, Cbz-C*^ar^***H**), 5.10 (s, 2H, Cbz-C**H_2_**-), 3.64 (t, ^3^*J* = 6.2 Hz, 2H, -C**H_2_**-OH), 3.21 (t, ^3^*J* = 6.8 Hz, 2H, -NH-C**H_2_**-), 1.62 – 1.51 (m, 4H, -NH-CH_2_-C**H_2_**-CH_2_-C**H_2_-**), 1.45 – 1.35 (m, 2H, -NH-CH_2_-CH_2_-C**H_2_**-).

^13^**C**{^1^**H**}**-NMR** (126 MHz, CDCl_3_):

δ [ppm] = 156.6 (Cbz-O**C**(O)NH-), 136.7 (Cbz-**C^1^**), 128.6 (Cbz-**C^3/3‘^**), 128.3 (Cbz-**C^2/2‘^**), 128.2 (Cbz-**C^4^**), 66.8 (Cbz-**C**H_2_-), 62.8 (-**C**H_2_-OH), 41.1 (-NH-**C**H_2_-), 32.4 (-NH-CH_2_-**C**H_2_-), 29.9 (-**C**H_2_-CH_2_-OH), 23.0 (-NH-CH_2_-CH_2_-**C**H_2_-).

#### 1.3.2.6 Benzyl (5-iodopentyl)carbamate (6)

Imidazole (4.08 g, 59.9 mmol, 2.0 eq.) and PPh_3_ (15.1 g, 57.4 mmol, 1.9 eq.) were suspended in DCM (70 mL) and stirred for 30 min at RT. To the resulting solution iodine (15.1 g, 59.4 mmol, 2.0 eq.) was added while cooling with an ice bath and the red suspension was stirred for 1 h. Cbz-protected alcohol **1** (7.02 g, 29.6 mmol, 1.0 eq.) was dissolved in DCM (50 mL) and added dropwise over 30 min. The reaction mixture was stirred overnight and complete conversion was determined by UP-LCMS. After removing the solvent under reduced pressure and purification of the crude by column chromatography (cyclohexane:ethyl acetate, 2:1, 10x12 cm) alkyl iodide **6** (6.86 g, 19.7 mmol, 69 %) was obtained as slight yellow liquid.

M: 347.20 g/mol | Yield: 69 %, 6.86 g, 19.7 mmol | C_13_H_18_INO_2_

**DC**: R*_f_* (cyclohexane/ethyl acetate, 2:1) = 0.53.

^1^**H-NMR** (500 MHz, CDCl_3_):

δ [ppm] = 7.40 – 7.27 (m, 5H, Cbz-C*^ar^***H**), 5.10 (s, 2H. Cbz-C**H_2_**-), 4.77 (s, 1H, Cbz-N**H**-), 3.24 – 3.14 (m, 4H, -NH-C**H_2_**, -C**H_2_**-OH), 1.88 – 1.79 (m, 2H, -NH-CH_2_-CH_2_-CH_2_-C**H_2_**-CH_2_-OH), 1.58 – 1.48 (m, 2H, -NH-CH_2_-C**H_2_**-CH_2_-CH_2_-CH_2_-OH), 1.47 – 1.38 (m, 2H, -NH-CH_2_-CH_2_-C**H_2_**-CH_2_-CH_2_-OH).

#### 1.3.2.7 Methyl (*S*)-3-(3-(5-(((benzyloxy)carbonyl)amino)pentyl)-4-hydroxyphenyl)-2-((tert butoxycarbonyl)-amino)propanoate (7a)

Zinc dust (preactivated, 23.0 mg, 352 µmol, 7.1 eq.), Pd(amphos)Cl_2_ (3.70 mg, 5.00 µmol, 0.1 eq.) and alkyl iodide **6** (43.0 mg, 124 µmol, 2.5 eq.) were added to a dried-out flask and the atmosphere was changed to argon for 15 minutes. Afterwards anhydrous DMF (200 µL) was added and degassed by bubbling argon through the suspension for 5 minutes. A solution (300 µL, 0.167 M, 1.0 eq.) of Boc-protected methyl ester **3a** (33.9 mg) in anhydrous DMF (482 µL) was degassed by bubbling argon through the solution for 5 minutes and then added. Afterwards the closed vial was stirred at 40 °C over night and after complete conversion determined by LCMS the reaction mixture was centrifuged and the supernatant purified by RP-HPLC. Freeze drying yielded Negishi product **7a** (16.5 mg, 32.0 µmol, 64 %).

M: 514.62 g/mol | Yield: 64 %, 16.5 mg, 32.0 µmol | C_28_H_38_N_2_O_7_

**DC**: R*_f_* (cyclohexane/ethyl acetate, 3:1) = 0.26.

**HPLC-MS** (ESI+):	*m*/*z* (experimental):	515.3080, t_R_ = 3.1 min.
*m*/*z* (calculated):	515.2752, [M+H]^+^

^1^**H-NMR** (500 MHz, methanol-*d*_4_):

δ [ppm] = 7.39 – 7.31 (m, 5H, Cbz-C*^ar^***H**), 6.88 (s, 1H, C^2^-**H**), 6.82 (d, ^3^*J* = 8.1 Hz, 1H, C^6^-**H**), 6.66 (d, ^3^*J* = 8.1 Hz, 1H, C^5^-**H**), 5.07 (s, 2H, Cbz-C**H_2_**-), 4.30 (dd, ^3^*J* = 8.4 Hz, ^3^*J* = 5.8 Hz, 1H, C^α^-**H**), 3.67 (s, 3H, -OC**H_3_**), 3.11 (t, ^3^*J* = 7.1 Hz, 2H, -NH-C**H_2_**-), 2.96 (dd, ^2^*J* = 13.9 Hz, ^3^*J* = 5.8 Hz, 1H, C^β^-**H**/H’), 2.80 (dd, ^2^*J* = 13.9 Hz, ^3^*J* = 8.4 Hz, 1H, C^β^-H/**H’**), 2.56 (t, ^3^*J* = 7.7 Hz, 2H, -C^3^-C**H_2_**-), 1.65 – 1.56 (m, 2H, -C^3^-CH_2_-C**H_2_**-), 1.56 – 1.49 (m, 2H, -NH-CH_2_-C**H_2_**-), 1.39 (s, 9H, Boc-**H**), 1.44 – 1.32 (m, 2H, -NH-CH_2_-CH_2_-C**H_2_**-).

^13^**C**{^1^**H**}**-NMR** (126 MHz, methanol-*d*_4_):

δ [ppm] = 174.4 (-**C**OOMe), 158.9 (Cbz-**C**ONH-), 157.8 (^t^BuO**C**ONH-), 155.1 (Tyr-**C**^4^), 138.5 (Cbz-**C**^1^), 132.0(Tyr-**C**^2^), 130.0 (Tyr-**C**^1^), 129.4 (Cbz-**C**^3/3’^), 128.9 (Cbz-**C**^4^), 128.7 (Cbz-**C**^2/2’^), 128.7 (Tyr-**C**^3^), 128.5 (Tyr-**C**^6^), 115.9 (Tyr-**C**^5^), 80.0 (-**C**(CH_3_)_3_), 67.3 (Cbz-**C**H_2_-), 56.8 (Tyr-**C**^α^), 52.5 (-**C**H_3_), 41.9 (-NH-**C**H_2_-), 38.0 (Tyr‑**C**^β^), 31.1 (Tyr-C^3^-**C**H_2_-), 30.8 (Tyr-C^3^-CH_2_-**C**H_2_-), 30.7 (-NH-CH_2_-**C**H_2_-), 28.7 (-C(**C**H_3_)_3_), 27.6 (-NH-CH_2_-CH_2_-**C**H_2_-).

#### 1.3.2.8 Methyl (*R*)-3-(3-(5-(((benzyloxy)carbonyl)amino)pentyl)-4-hydroxyphenyl)-2-((tert-butoxycarbonyl)-amino)propanoate (7b)

Zinc dust (preactivated, 201 mg, 3.07 mmol, 2.0 eq.), Pd(amphos)Cl_2_ (109 mg, 0.15 mmol, 0.1 eq.) and alkyl iodide **6** (1.13 g, 3.03 mmol, 2.0 eq.) were added to a dried-out flask and the atmosphere was changed to argon for 50 minutes. Afterwards anhydrous DMF (6.00 mL) was added and degassed by bubbling argon through the suspension for 30 minutes. A solution (9.00 mL, 0.167 M, 1.0 eq.) of Boc-protected methyl ester **3b** (651 mg) in anhydrous DMF (9.25 mL) was degassed by bubbling argon through the solution for 30 minutes and then added. Afterwards the closed vial was stirred at 40 °C over night and after complete conversion determined by LCMS the reaction mixture was centrifuged and the supernatant purified by RP-HPLC. Freeze drying yielded Negishi product **7b** (562 mg, 1.09 mmol, 72 %).

M: 514.62 g/mol | Yield: 72 %, 562 mg, 1.09 mmol | C_28_H_38_N_2_O_7_

**UHPLC-MS** (ESI+):	*m*/*z* (experimental):	515.2670, t_R_ = 3.2 min.
*m*/*z* (calculated):	515.2752, [M+H]^+^

^1^**H-NMR** (600 MHz, CDCl_3_):

δ [ppm] = 7.48 – 7.28 (m, 5H, Cbz-C*^ar^***H**), 6.87 – 6.74 (m, 2H, C^2^-**H** and C^6^-**H**), 6.66 (d, ^3^*J* = 7.9 Hz, 1H, C^5^-**H**), 5.10 (s, 2H, Cbz-C**H_2_**-), 4.52 (dd, ^3^*J* = 6.7 Hz, ^3^*J* = 6.6 Hz, 1H, C^α^-**H**), 3.69 (s, 3H, -OC**H_3_**), 3.18 (t, ^3^*J* = 8.5 Hz, 2H, -NH-C**H_2_**-), 3.06 – 2.90 (m, 2H, C^β^-**H_2_**), 2.70 – 2.46 (m, 2H, , -C^3^-C**H_2_**-), 1.67 – 1.56 (m, 2H, -C^3^-CH_2_-C**H_2_**-), 1.56 – 1.48 (m, 2H, -NH-CH_2_-C**H_2_**-), 1.42 (s, 9H, Boc-**H**), 1.46 – 1.28 (m, 2H, -NH-CH_2_-CH_2_-C**H_2_**-).

^13^**C**{^1^**H**}**-NMR** (151 MHz, CDCl_3_):

δ [ppm] = 172.7 (-**C**OOMe), 156.7 (Cbz-**C**ONH-), 155.3 (^t^BuO**C**ONH-), 153.0 (Tyr-**C**^4^), 136.7 (Cbz-**C**^1^), 131.2 (Tyr-**C**^2^), 128.7 (Cbz-**C**^3/3’^), 128.2 (Tyr-**C**^1^), 128.2 (Cbz-**C**^2/2’^), 127.9 (Tyr-**C**^6^), 127.9 (Cbz-**C**^4^), 127.8 (Tyr-**C**^3^), 115.6 (Tyr-**C**^5^), 80.1 (-**C**(CH_3_)_3_), 66.8 (Cbz-**C**H_2_-), 54.7 (Tyr-**C**^α^), 52.3 (-**C**H_3_), 41.0 (-NH-**C**H_2_-), 37.6 (Tyr‑**C**^β^), 30.0 (Tyr-C^3^-**C**H_2_-), 29.8 (Tyr-C^3^-CH_2_-**C**H_2_-), 29.4 (-NH-CH_2_-**C**H_2_-), 28.5 (-C(**C**H_3_)_3_), 26.4 (-NH-CH_2_-CH_2_-**C**H_2_-).

#### 1.3.2.9 Allyl (5-hydroxypentyl)carbamate (8)

5-Aminopentan-1-ol **(4)** (2.25 g, 21.8 mmol, 1.0 eq.) and NaHCO_3_ (6.28 g, 74.8 mmol, 3.5 eq.) were suspended in distilled water (15 mL) and a solution of Alloc-Cl (3.20 mL, 30.1 mmol, 1.4 eq.) in THF (10 mL) was added dropwise under cooling with an ice bath. The reaction mixture was stirred overnight, while slowly warming to RT. Reaction progress was monitored by TLC and after complete consumption of starting material the suspension was diluted with water (100 mL) and ethyl acetate (25 mL). The aqueous layer was extracted with ethyl acetate (3x50 mL) and the combined organic layers washed with HCl (2x25 mL, 0.2 M, aq.) and brine (2x20 mL), and dried over MgSO_4_. After removing the solvent under reduced pressure Alloc-protected alcohol **8** (3.21 g, 17.1 mmol, 80 %) was obtained as colorless liquid.

M: 187.24 g/mol | Yield: 80 %, 3.21 g, 17.1 mmol | C_9_H_17_NO_3_

**DC**: R*_f_* (cyclohexane/ethyl acetate, 2:1) = 0.5.

^1^**H-NMR** (500 MHz, CDCl_3_):

δ [ppm] = 5.91 (ddt, ^3^*J*_trans_ = 16.4 Hz, ^3^*J*_cis_ = 10.7 Hz, ^3^*J* = 5.7 Hz, 1H, H_2_C=C**H**-CH_2_-), 5.29 (d, ^3^*J*_trans_ = 17.4 Hz, 1H, **H_2_**C=CH-CH_2_-), 5.20 (d, ^3^*J*_cis_ = 10.2 Hz, 1H, **H_2_**C=CH-CH_2_-), 4.55 (d, ^3^*J* = 5.7 Hz, 2H, H_2_C=CH-C**H_2_**-), 3.64 (t, ^3^*J* = 6.5 Hz, 2H, -C**H_2_**-OH), 3.26 – 3.12 (m, 2H, Alloc-NH-C**H_2_**-), 1.63 – 1.56 (m, 2H, -C**H_2_**-CH_2_-OH), 1.56 – 1.49 (m, 2H, Alloc-NH-CH_2_-C**H_2_**-), 1.46 – 1.35 (m, 2H, Alloc-NH-CH_2_-CH_2_-C**H_2_**-).

^13^**C**{^1^**H**}**-NMR** (126 MHz, CDCl_3_):

δ [ppm] = 156.5 (H_2_C=CH-CH_2_-**C**(O)O-NH-), 133.1 (H_2_**C**=CH-CH_2_-) , 117.8 (H_2_C=**C**H-CH_2_-), 65.6 (H_2_C=CH-**C**H_2_-), 62.8 (-**C**H_2_-OH), 41.0 (-NH-**C**H_2_-), 32.4 (-NH-CH_2_-**C**H_2_-), 29.9 (-**C**H_2_-CH_2_-OH), 23.0 (-NH-CH_2_-CH_2_-**C**H_2_-).

#### 1.3.2.10 Methyl (*S*)-3-(4-((5-(((allyloxy)carbonyl)amino)pentyl)oxy)-3-(5-(((benzyloxy) carbonyl)amino)-pentyl)phenyl)-2-((tert-butoxycarbonyl)amino)propanoate (9a)

Under argon atmosphere Negishi product **7a** (59.0 mg, 115 µmol, 1.0 eq.), ADDP (59 mg, 234 µmol, 2.0 eq.) and Alloc-protected alcohol **8** (46 mg, 246 µmol, 2.1 eq.) were dissolved in anhydrous THF (5.00 mL) and degassed by *freeze-pump-thaw*. P(*n*-Bu)_3_ (59 µL, 236 µmol, 2.1 eq.) was added and the yellow solution stirred for 1 h at 60 °C. After removing the solvent under reduced pressure, the crude was dissolved in a mixture of acetonitrile, water and DMF and purified by RP-HPLC. Freeze drying yielded Mitsunobu product **9a** (63.5 mg, 92.8 µmol, 81 %) as colorless solid.

M: 683.84 g/mol | Yield: 81 %, 63.5 mg, 92.8 µmol | C_37_H_53_N_3_O_9_

**UHPLC-MS** (ESI+):	*m*/*z* (experimental):	684.3751, t_R_ = 3.6 min.
*m*/*z* (calculated):	684.3855, [M+H]^+^

^1^**H-NMR** (500 MHz, methanol-*d*_4_):

δ [ppm] = 7.41 – 7.21 (m, 5H, Cbz-C*^ar^***H**), 6.98 – 6.90 (m, 2H, C^2^-**H** and C^6^-**H**), 6.78 (d, ^3^*J* = 8.1 Hz, 1H, C^5^-**H**), 5.91 (ddt, ^2^*J*_trans_ = 16.2 Hz, ^2^*J*_cis_ = 10.6 Hz, ^3^*J* = 5.4 Hz, 1H, H_2_C=C**H**-CH_2_-), 5.28 (d, ^2^*J*_trans_ = 17.2 Hz, 1H, **H**_2_C=CH-CH_2_-), 5.15 (d, ^2^*J*_cis_ = 10.6 Hz, 1H, **H**_2_C=CH-CH_2_-), 5.06 (s, 2H, Cbz-C**H_2_**-), 4.50 (d, ^3^*J* = 5.4 Hz, 2H, H_2_C=CH-C**H_2_**-), 4.30 (dd, ^3^*J* = 8.1 Hz, ^3^*J* = 5.9 Hz, 1H, C^α^-**H**), 3.94 (t, ^3^*J* = 6.2 Hz, 2H, -C**H_2_**-O-Tyr), 3.67 (s, 3H, -OC**H_3_**), 3.15 – 3.07 (m, 4H, Alloc-NH-C**H_2_**- and Cbz-NH-C**H_2_**-), 2.98 (dd, ^2^*J* = 13.8 Hz, ^3^*J* = 5.8 Hz, 1H, C^β^-H/**H’**), 2.81 (dd, ^2^*J* = 13.9 Hz, ^3^*J* = 8.6 Hz, 1H, C^β^-**H**/H’), 2.57 (t, ^3^*J* = 7.7 Hz, 2H, -C^3^-C**H_2_**-), 1.84 – 1.76 (m, 2H, -C**H_2_**-CH_2_-O-Tyr), 1.64 – 1.46 (m, 8H, Alloc-NH-CH_2_-C**H_2_**-C**H_2_**-, -C^3^-CH_2_-C**H_2_**-, Cbz-NH-CH_2_-C**H_2_**-), 1.38 (s, 9H, Boc-**H**), 1.42 – 1.24 (m, 2H, Cbz-NH-CH_2_-CH_2_-C**H_2_**).

^13^**C**{^1^**H**}**-NMR** (126 MHz, methanol-*d*_4_):

δ [ppm] = 174.4 (-**C**OOMe), 158.9 (Cbz-**C**ONH-), 158.8 (Alloc-O**C**(O)NH-), 157.8 (tBuO**C**ONH-), 157.2 (Tyr-**C**^4^), 138.5 (Cbz-**C**^1^), 134.6 (H_2_C=**C**H-CH_2_-), 132.0 (Tyr-**C**^3^), 131.8 (Tyr-**C**^2^), 129.6 (Tyr-**C**^1^), 129.5 (Cbz-**C**^3/3’^), 128.9 (Cbz-**C**^4^), 128.8 (Tyr-**C**^6^), 128.7 (Cbz-**C**^2/2’^), 117.4 (H_2_**C**=CH-CH_2_-), 112.2 (Tyr-**C**^5^), 80.6 (-**C**(CH_3_)_3_), 68.7 (-**C**H_2_-O-Tyr), 67.3 (Cbz-**C**H_2_-), 66.2 (H_2_C=CH-**C**H_2_-), 56.8 (Tyr-**C**^α^), 52.6 (-**C**H_3_), 41.9 (Cbz-NH-**C**H_2_-), 41.7 (Alloc-NH-**C**H_2_-), 38.0 (Tyr-**C**^β^), 31.3 (-C^3^-**C**H_2_-), 30.9 (-C^3^-CH_2_-**C**H_2_-), 30.8 (Cbz-NH-CH_2_-**C**H_2_-), 30.7 (Alloc-NH-CH_2_-**C**H_2_-), 30.2 (-**C**H_2_-CH_2_-O-Tyr) 28.7 (-C(**C**H_3_)_3_), 27.7 (Cbz-NH-CH_2_-CH_2_-**C**H_2_), 24.6 (**C**H_2_-CH_2_-CH_2_-O-Tyr).

#### 1.3.2.11 Methyl (*R*)-3-(4-((5-(((allyloxy)carbonyl)amino)pentyl)oxy)-3-(5-(((benzyloxy) carbonyl)amino)-pentyl)phenyl)-2-((tert-butoxycarbonyl)amino)propanoate (9b)

Under argon atmosphere Negishi product **7b** (562 mg, 1.50 mmol, 1.0 eq.), ADDP (767 mg, 3.04 mmol, 2.0 eq.) and Alloc-protected alcohol **8** (573 mg, 3.06 mmol, 2.0 eq.) were dissolved in anhydrous THF (50.0 mL) and degassed by *freeze-pump-thaw*. P(*n*-Bu)_3_ (0.75 mL, 3.00 mmol, 2.0 eq.) was added and the yellow solution stirred for 1 h at 60 °C. After removing the solvent under reduced pressure, the crude was dissolved in a mixture of acetonitrile, water and DMF and purified by RP-HPLC. Freeze drying yielded Mitsunobu product **9b** (529 mg, 0.77 mmol, 52 %) as colorless solid.

M: 683.84 g/mol | Yield: 52 %, 529 mg, 0.77 mmol | C_37_H_53_N_3_O_9_

**HPLC-MS** (ESI+):	*m*/*z* (experimental):	684.3073, t_R_ = 3.6 min.
*m*/*z* (calculated):	684.3855, [M+H]^+^

^1^**H-NMR** (600 MHz, methanol-*d*_4_):

δ [ppm] = 7.45 – 7.21 (m, 5H, Cbz-C*^ar^***H**), 6.98 – 6.89 (m, 2H, C^2^-**H** and C^6^-**H**), 6.78 (d, ^3^*J* = 8.2 Hz, 1H, C^5^-**H**), 5.91 (ddt, ^2^*J*_trans_ = 16.2 Hz, ^2^*J*_cis_ = 10.6 Hz, ^3^*J* = 5.4 Hz, 1H, H_2_C=C**H**-CH_2_-), 5.28 (d, ^2^*J*_trans_ = 17.3 Hz, 1H, **H**_2_C=CH-CH_2_-), 5.15 (d, ^2^*J*_cis_ = 10.5 Hz, 1H, **H**_2_C=CH-CH_2_-), 5.06 (s, 2H, Cbz-C**H_2_**-), 4.50 (d, ^3^*J* = 5.2 Hz, 2H, H_2_C=CH-C**H_2_**-), 4.30 (dd, ^3^*J* = 8.5 Hz, ^3^*J* = 5.8 Hz, 1H, C^α^-**H**), 3.94 (t, ^3^*J* = 6.2 Hz, 2H, -C**H_2_**-O-Tyr), 3.67 (s, 3H, -OC**H_3_**), 3.16 – 3.06 (m, 4H, Alloc-NH-C**H_2_**- and Cbz-NH-C**H_2_**), 2.98 (dd, ^2^*J* = 13.8, ^3^*J* = 5.8 Hz, 1H, C^β^-H/**H’**), 2.81 (dd, ^2^*J* = 13.8, ^3^*J* = 8.6 Hz, 1H, C^β^-**H**/H’), 2.57 (t, ^3^*J* = 7.7 Hz, 2H, -C^3^-C**H_2_**-), 1.87 – 1.74 (m, 2H, -C**H_2_**-CH_2_-O-Tyr), 1.65 – 1.47 (m, 8H, Alloc-NH-CH_2_-C**H_2_**-C**H_2_**-, -C^3^-CH_2_-C**H_2_**-, Cbz-NH-CH_2_-C**H_2_**-), 1.38 (s, 9H, Boc-**H**), 1.44 – 1.27 (m, 2H, Cbz-NH-CH_2_-CH_2_-C**H_2_**).

^13^**C**{^1^**H**}**-NMR** (151 MHz, methanol-*d*_4_):

δ [ppm] = 174.4 (-**C**OOMe), 158.9 (Cbz-**C**ONH-), 158.8 (Alloc-O**C**(O)NH-), 157.8 (tBuO**C**ONH-), 157.2 (Tyr-**C**^4^), 138.5 (Cbz-**C**^1^), 134.6 (H_2_C=**C**H-CH_2_-), 132.0 (Tyr-**C**^3^), 131.8 (Tyr-**C**^2^), 129.6 (Tyr-**C**^1^), 129.5 (Cbz-**C**^3/3’^), 128.9 (Cbz-**C**^4^), 128.8 (Tyr-**C**^6^), 128.7 (Cbz-**C**^2/2’^), 117.3 (H_2_**C**=CH-CH_2_-), 112.2 (Tyr-**C**^5^), 80.6 (-**C**(CH_3_)_3_), 68.7 (-**C**H_2_-O-Tyr), 67.3 (Cbz-**C**H_2_-), 66.2 (H_2_C=CH-**C**H_2_-), 56.8 (Tyr-**C**^α^), 52.6 (-**C**H_3_), 41.9 (Cbz-NH-**C**H_2_-), 41.7 (Alloc-NH-**C**H_2_-), 38.0 (Tyr-**C**^β^), 31.3 (-C^3^-**C**H_2_-), 30.9 (-C^3^-CH_2_-**C**H_2_-), 30.8 (Cbz-NH-CH_2_-**C**H_2_-), 30.7 (Alloc-NH-CH_2_-**C**H_2_-), 30.2 (-**C**H_2_-CH_2_-O-Tyr), 28.7 (‑C(**C**H_3_)_3_), 27.7 (Cbz-NH-CH_2_-CH_2_-**C**H_2_), 24.6 (**C**H_2_-CH_2_-CH_2_-O-Tyr).

#### 1.3.2.12 Methyl (*S*)-3-(3-(5-(((benzyloxy)carbonyl)amino)pentyl)-4-((5-((4,5-dihydro-1H-imidazol-2-yl)amino-)pentyl)oxy)phenyl)-2-((2,4,6-trimethylphenyl)sulfonamido) propanoate (10a)

Mitsunobu product **9a** (434 mg, 0.63 mmol, 1.0 eq.) was dissolved in DCM (5 mL) and HCl in dioxane (4 M, 1.60 mL, 6.35 mmol, 10 eq.) was added and the solution stirred at RT until complete conversion determined by LCMS. Subsequently volatile compounds were removed under HV and the residue dissolved in a mixture of DCM and DMF (4.4 mL, 10:1, v:v). DIPEA (324 µL, 1.90 mmol, 3.0 eq.) and Mesitylene sulfochloride (172 mg, 0.79 mmol, 1.2 eq.) were added and the reaction mixture stirred at RT until complete conversion determined by LCMS. Afterwards volatile compounds were removed under HV and the residue dissolved in DCM (8 mL). Morpholine (553 µL, 6.35 mmol, 10 eq.) and Tetrakis(triphenylphosphine)palladium(0) (73.5 mg, 0.06 mmol, 0.1 eq.) were added and the solution stirred at RT until complete conversion determined by LCMS. Subsequently volatile compounds were removed under HV and the residue suspended in a solution of 2-methylthio-2-imidazoline hydroiodide (0.18 M in MeOH/NEt_3_, (1:1, v:v), 14 mL, 4.0 eq.). The reaction mixture was stirred at 80 °C for 4 h until complete conversion determined by LCMS. The suspension was concentrated in vacuum and a mixture of acetonitrile, water and DMF (2.1:1:1.4, v:v:v) was added. After centrifugation the supernatant was purified by RP-HPLC. Freeze-drying yielded sulfonamide **10a** (426 mg, 0.57 mmol, 90 %) as colorless solid.

M: 749.97 g/mol | Yield: 90 %, 426 mg, 0.57 mmol | C_40_H_55_N_5_O_7_S

**UHPLC-MS** (ESI+):	*m*/*z* (experimental):	750.4370, t_R_ = 2.9 min.
*m*/*z* (calculated):	750.3895, [M+H]^+^

^1^**H-NMR** (500 MHz, methanol-*d*_4_):

δ [ppm] = 8.56 (s broad, 1H), 7.43 – 7.22 (m, 5H, Cbz-C*^ar^***H**), 6.88 (s, 2H, Ar-C^3/3’^**H**), 6.82 (dd, ^3^*J* = 8.3 Hz, ^4^*J* = 2.3 Hz, 1H, Tyr-C^6^-**H**), 6.77 (d, ^4^*J* = 2.2 Hz, 1H, Tyr-C^2^-**H**), 6.67 (d, ^3^*J* = 8.3 Hz, 1H Tyr-C^5^-**H**), 5.06 (s, 2H, Cbz-C**H_2_**-), 3.94 (t, ^3^*J* = 6.1 Hz, 2H, -C**H_2_**-O-Tyr), 3.87 (dd, ^3^*J* = 8.9 Hz, ^3^*J* = 6.1 Hz, 1H, Tyr-C^α^-**H**), 3.67 (s, 4H, Im-C^3/3’^**H_2_**), 3.44 (s, 3H, -OC**H_3_**), 3.21 (t, ^3^*J* = 7.0 Hz, 2H, Im-NH-C**H_2_**-), 3.10 (t, ^3^*J* = 7.1 Hz, 2H, Cbz-NH-C**H_2_**-), 2.89 (dd, ^2^*J* = 13.8 Hz, ^3^*J* = 6.1 Hz, 1H, C^β^-H/**H’**), 2.72 (dd, ^2^*J* = 13.8 Hz, ^3^*J* = 8.9 Hz, 1H, C^β^-**H**/H’), 2.57 – 2.40 (m, 2H, Tyr-C^3^-C**H_2_**-), 2.47 (s, 6H, Ar-C^2/2’^-C**H_3_**), 2.26 (s, 3H, Ar-C^4^-C**H_3_**), 1.86 – 1.78 (m, 2H, -C**H_2_**-CH_2_-O-Tyr), 1.72 – 1.62 (m, 2H, Im-NH-CH_2_-C**H_2_**), 1.62 – 1.46 (m, 6H, -C**H_2_**-CH_2_-CH_2_-O-Tyr, Tyr-C^3^-CH_2_-C**H_2_**-CH_2_-C**H_2_**-), 1.37 – 1.27 (m, 2H, Cbz-NH-CH_2_-CH_2_-C**H_2_**).

^13^**C**{^1^**H**}**-NMR** (126 MHz, methanol-*d*_4_):

δ [ppm] = 173.6 (-**C**OOMe), 161.2 (Im-**C^1^**), 158.9 (Cbz-**C**ONH-), 157.1 (Tyr-**C**^4^), 143.4 (Ar-**C^1^**), 140.5 (Ar-**C^2/2’^**), 138.5 (Cbz-**C**^1^), 135.1 (Ar-**C^4^**), 132.8 (Ar-**C^3/3’^**), 131.9 (Tyr-**C**^3^), 131.5 (Tyr-**C**^2^), 129.5 (Cbz-**C**^3/3’^), 129.1 (Cbz-**C**^4^), 128.9 (Tyr-**C**^1^), 128.7 (Tyr-**C**^6^), 128.6 (Cbz-**C**^2/2’^), 112.1 (Tyr-**C**^5^), 68.6 (-**C**H_2_-O-Tyr), 67.3 (Cbz-**C**H_2_-), 58.9 (Tyr-**C**^α^), 52.5 (-**C**H_3_), 44.0 (Im-**C^3/3’^**), 43.8 (Im-NH-**C**H_2_-), 41.8 (Cbz-NH-**C**H_2_-), 38.7 (Tyr-**C**^β^), 31.2 (Tyr-C^3^-**C**H_2_-), 30.8 (Tyr-C^3^-CH_2_-**C**H_2_-), 30.7 (Cbz-NH-CH_2_-**C**H_2_-), 30.1 (-**C**H_2_-CH_2_-O-Tyr), 29.9 (Im-NH-CH_2_-**C**H_2_-), 27.7 (Cbz-NH-CH_2_-CH_2_-**C**H_2_), 24.6 (**C**H_2_-CH_2_-CH_2_-O-Tyr), 23.2 (Ar-C^2/2’^-**C**H_3_), 21.0 (Ar-C^4^-**C**H_3_).

#### 1.3.2.13 Methyl (*R*)-3-(3-(5-(((benzyloxy)carbonyl)amino)pentyl)-4-((5-((4,5-dihydro-1H-imidazol-2-yl)amino-)pentyl)oxy)phenyl)-2-((2,4,6-trimethylphenyl)sulfonamido) propanoate (10b)

Mitsunobu product **9b** (529 mg, 0.77 mmol, 1.0 eq.) was dissolved in DCM (5 mL) and HCl in dioxane (4 M, 1.90 mL, 7.74 mmol, 10 eq.) was added and the solution stirred at RT until complete conversion determined by LCMS. Subsequently volatile compounds were removed under HV and the residue dissolved in a mixture of DCM and DMF (4.4 mL, 10:1, v:v). DIPEA (395 µL, 2.32 mmol, 3.0 eq.) and Mesitylene sulfochloride (213 mg, 0.97 mmol, 1.3 eq.) were added and the reaction mixture stirred at RT until complete conversion determined by LCMS. Afterwards volatile compounds were removed under HV and the residue dissolved in DCM (8 mL). Morpholine (674 µL, 7.74 mmol, 10 eq.) and Tetrakis(triphenylphosphine)palladium(0) (90.5 mg, 0.08 mmol, 0.1 eq.) were added and the solution stirred at RT until complete conversion determined by LCMS. Subsequently volatile compounds were removed under HV and the residue suspended in a solution of 2-methylthio-2-imidazoline hydroiodide (0.18 M in MeOH/NEt_3_, (1:1, v:v), 17.2 mL, 4.1 eq.). The reaction mixture was stirred at 80 °C for 4 h until complete conversion determined by LCMS. The suspension was concentrated in vacuum and a mixture of acetonitrile, water and DMF (5:1:2, v:v:v) was added. After centrifugation the supernatant was purified by RP-HPLC. Freeze-drying yielded sulfonamide **10b** (404 mg, 0.54 mmol, 70 %) as colorless solid.

M: 749.97 g/mol | Yield: 70 %, 404 mg, 0.54 mmol | C_40_H_55_N_5_O_7_S

**HPLC-MS** (ESI+):	*m*/*z* (experimental):	750.2755, t_R_ = 2.8 min.
*m*/*z* (calculated):	750.3895, [M+H]^+^

^1^**H-NMR** (600 MHz, methanol-*d*_4_):

δ [ppm] = 7.37 – 7.26 (m, 5H, Cbz-C*^ar^***H**), 6.88 (s, 2H, Ar-C^3/3’^**H**), 6.81 (dd, ^3^*J* = 8.2 Hz, ^4^*J* = 2.3 Hz, 1H, Tyr-C^6^-**H**), 6.76 (d, ^4^*J* = 2.5 Hz, 1H, Tyr-C^2^-**H**), 6.66 (d, ^3^*J* = 8.3 Hz, 1H, Tyr-C^5^-**H**), 5.06 (s, 2H, Cbz-C**H_2_**-), 3.93 (t, ^3^*J* = 6.1 Hz, 2H, -C**H_2_**-O-Tyr), 3.87 (dd, ^3^*J* = 8.9 Hz, ^3^*J* = 6.1 Hz, 1H, Tyr-C^α^-**H**), 3.66 (s, 4H, Im-C^3/3’^**H_2_**), 3.44 (s, 3H, -OC**H_3_**), 3.20 (t, ^3^*J* = 7.1 Hz, 2H, Im-NH-C**H_2_**-), 3.09 (t, ^3^*J* = 7.1 Hz, 2H, Cbz-NH-C**H_2_**-), 2.89 (dd, ^2^*J* = 13.8 Hz, ^3^*J* = 6.1 Hz, 1H, C^β^-H/**H’**), 2.72 (dd, ^2^*J* = 13.8 Hz, ^3^*J* = 8.9 Hz, 1H, C^β^-**H**/H’), 2.47 (s, 6H, Ar-C^2/2’^-C**H_3_**), 2.57 – 2.39 (m, 2H, Tyr-C^3^-C**H_2_**-), 2.26 (s, 3H, Ar-C^4^-C**H_3_**), 1.86 – 1.79 (m, 2H, -C**H_2_**-CH_2_-O-Tyr), 1.70 – 1.63 (m, 2H, Im-NH-CH_2_-C**H_2_**), 1.60 – 1.47 (m, 6H, -C**H_2_**-CH_2_-CH_2_-O-Tyr, Tyr-C^3^-CH_2_-C**H_2_**-CH_2_-C**H_2_**-), 1.36 – 1.27 (m, 2H, Cbz-NH-CH_2_-CH_2_-C**H_2_**).

^13^**C**{^1^**H**}**-NMR** (151 MHz, methanol-*d*_4_):

δ [ppm] = 173.6 (-**C**OOMe), 161.2 (Im-**C^1^**), 158.9 (Cbz-**C**ONH-), 157.1 (Tyr-**C**^4^), 143.4 (Ar-**C^1^**), 140.5 (Ar-**C^2/2’^**), 138.5 (Cbz-**C**^1^), 135.1 (Ar-**C^4^**), 132.8 (Ar-**C^3/3’^**), 131.9 (Tyr-**C**^3^), 131.5 (Tyr-**C**^2^), 129.5 (Cbz-**C**^3/3’^), 129.1 (Cbz-**C**^4^), 128.9 (Tyr-**C**^1^), 128.7 (Tyr-**C**^6^), 128.6 (Cbz-**C**^2/2’^), 112.1 (Tyr-**C**^5^), 68.6 (-**C**H_2_-O-Tyr), 67.3 (Cbz-**C**H_2_-), 58.9 (Tyr-**C**^α^), 52.6 (-**C**H_3_), 44.0 (Im-**C^3/3’^**), 43.8 (Im-NH-**C**H_2_-), 41.8 (Cbz-NH-**C**H_2_-), 38.7 (Tyr-**C**^β^), 31.2 (Tyr-C^3^-**C**H_2_-), 30.8 (Tyr-C^3^-CH_2_-**C**H_2_-), 30.7 (Cbz-NH-CH_2_-**C**H_2_-), 30.1 (-**C**H_2_-CH_2_-O-Tyr), 29.9 (Im-NH-CH_2_-**C**H_2_-), 27.7 (Cbz-NH-CH_2_-CH_2_-**C**H_2_), 24.5 (**C**H_2_-CH_2_-CH_2_-O-Tyr), 23.1 (Ar-C^2/2’^-**C**H_3_), 21.0 (Ar-C^4^-**C**H_3_).

#### 1.3.2.14 2-(2-(2-azidoethoxy)ethoxy)ethan-1-amine (12)

Azide **S3** (9.35 g, 46.7 mmol, 1.0 eq.) was dissolved in a mixture of diethyl ether, 1 M HCl and THF (150 mL, 5:5:1, v:v:v) and triphenylphosphine dissolved in diethyl ether (75 mL) was added dropwise over 3 h. The resulting suspension was stirred at RT for 16 h, followed by separation of the phases. The organic layer was extracted with 4 M HCl (aq., 2x80 mL) and the combined aqueous phases washed with diethyl ether (5x100 mL). Afterwards the pH-value was changed to pH = 14 by addition of NaOH-pellets, followed by extraction of the aqueous phase with DCM (4x50 mL). After drying the combined organic layers with MgSO_4_, filtration and removing the solvent under reduced pressure, azido amine **12** (6.56 g, 37.7 mmol, 81 %) was obtained as slight yellow liquid.

M: 174.20 g/mol | Yield: 81 %, 6.56 g, 37.7 mmol | C_6_H_14_N_4_O_2_

^1^**H-NMR** (500 MHz, CDCl_3_):

δ [ppm] = 3.71 – 3.61 (m, 6H, N_3_-CH_2_-C**H_2_**-O-C**H_2_**-C**H_2_**-O-), 3.51 (t, ^3^*J* = 6.1 Hz, 2H, -C**H_2_**-CH_2_-NH_2_), 3.39 (t, ^3^*J* = 6.2 Hz, 2H, N_3_-C**H_2_**-), 2.89 – 2.83 (m, 2H, -C**H_2_**-NH_2_), 1.49 (s, 2H, -N**H_2_**).

#### 1.3.2.15 4-((2-(2-(2-azidoethoxy)ethoxy)ethyl)amino)-4-oxobutanoic acid (13)

Azido amine **12** (2.93 g, 16.8 mmol, 1.0 eq.) was dissolved in acetonitrile (175 mL) and succinic anhydride (2.02 g, 20.1 mmol, 1.2 eq.) dissolved in acetonitrile (175 mL) was added dropwise over 3.5 h. Afterwards the reaction mixture was stirred at RT for 2 d. Removing the solvent under reduced pressure and purification of the crude by automatic NP-MPLC yielded azido acid **13** (3.55 g, 12.9 mmol, 77 %) as slight yellow oil.

M: 274.28 g/mol | Yield: 77 %, 3.55 g, 12.9 mmol | C_6_H_14_N_4_O_2_

^1^**H-NMR** (500 MHz, CDCl_3_):

δ [ppm] = 6.43 (s, 1H, -N**H**CO-), 3.76 – 3.60 (m, 6H, N_3_-CH_2_-C**H_2_**-O-C**H_2_**-C**H_2_**-O-), 3.56 (t, ^3^*J* = 5.8 Hz, 2H, -C**H_2_**-CH_2_-NH_2_), 3.50 – 3.44 (m, 2H, -C**H_2_**-NHCO-), 3.41 (t, ^3^*J* = 5.6 Hz, 2H, N_3_-C**H_2_**-), 2.68 (t, ^3^*J* = 5.1 Hz, 2H, -NHCO-C**H_2_**-), 2.52 (t, ^3^*J* = 5.2 Hz, 2H, -C**H_2_**-COOH).

#### 1.3.2.16 Methyl (*S*)-3-(3-(1-azido-10,13-dioxo-3,6-dioxa-9,14-diazanonadecan-19-yl)-4-((5-((4,5-dihydro-1H-imidazol-2-yl)amino)pentyl)oxy)phenyl)-2-((2,4,6-trimethylphenyl)sulfon amido)propanoate (14a)

Sulfonamide **10a** (426 mg, 0.57 mmol, 1.0 eq.) was dissolved in MeOH (30 mL) and Pd/C (10 %, 61.7 mg, 0.1 eq.) was added, followed by removal of the atmosphere using a waterjet pump (200 mbar) and enrichment with H_2_ (800 mbar). The resulting suspension was stirred at RT for 1 h until complete conversion determined by LCMS and filtered through celite. The solvent was removed under reduced pressure giving the crude amine (343 mg). Azido acid **13** (317 mg, 1.16 mmol, 2.1 eq.) was dissolved in dry DMF (5 mL) followed by addition of DIPEA (390 µL, 2.23 mmol, 4.0 eq.) and HATU (320 mg, 0.84 mmol, 1.5 eq.). To this yellow solution a solution of the crude amine in dry DMF (2 mL) was added and the reaction mixture stirred for 2 d at RT. Purification by RP-HPLC yielded conjugable RGD-mimetic precursor **14a** (238 mg, 0.27 mmol, 49 %).

M: 872.10 g/mol | Yield: 49 %, 238 mg, 0.27 mmol | C_42_H_65_N_9_O_9_S

**HPLC-MS** (ESI+):	*m*/*z* (experimental):	437.0357, t_R_ = 7.0 min.
*m*/*z* (calculated):	436.7385, [M+2H]^2+^

^1^**H-NMR** (500 MHz, DMSO-*d*_6_):

δ [ppm] = 8.25 (d, ^3^*J* = 9.4 Hz, 1H, Tyr-N**H**-SO_2_-), 8.22 (t, ^3^*J* = 5.9 Hz, 1H, Im-N**H**-), 7.86 (t, ^3^*J* = 5.5 Hz, 1H, -O-CH_2_-CH_2_-N**H**CO-), 7.79 (t, ^3^*J* = 5.6 Hz, 1H, -O-CH_2_-CH_2_-NHCO-CH_2_-CH_2_-CON**H**‑), 6.88 (s, 2H, Ar-C^3/3‘^**H**), 6.83 (d, ^3^*J* = 8.2 Hz, 1H, Tyr-C^6^-**H**), 6.75 (s, 1H, Tyr-C^2^-**H**), 6.68 (d, ^3^*J* = 8.4 Hz, 1H, Tyr-C^5^-**H**), 3.89 (t, ^3^*J* = 6.3 Hz, 1H, -C**H_2_**-O-Tyr), 3.75 (ddd, ^3^*J* = 9.1 Hz, ^3^*J* = 8.9 Hz, ^3^*J* = 6.3 Hz, 1H, Tyr-C^α^-**H**), 3.63 – 3.48 (m, 10H, N_3_-CH_2_-C**H_2_**-O-C**H_2_**-C**H_2_**-, Im-C^3/3’^**H_2_**), 3.45 – 3.37 (m, 4H, N_3_-C**H_2_**-, -O-C**H_2_**-CH_2_-NHCO-), 3.33 (s, 3H, -OC**H_3_**), 3.23 – 3.09 (m, 4H, -O-CH_2_-C**H_2_**-NHCO‑, -CH_2_-C**H_2_**-NHCO-), 3.00 (dt, ^3^*J* = 6.7 Hz, ^3^*J* = 6.7 Hz, 2H, Im-NH-C**H_2_**-), 2.80 (dd, ^2^*J* = 13.8 Hz, ^3^*J* = 6.1 Hz, 1H, C^β^-**H**/H’), 2.66 (dd, ^2^*J* = 13.8 Hz, ^3^*J* = 9.2 Hz, 1H, C^β^-H/**H’**), 2.41 (s, 6H, Ar-C^2/2‘^-C**H_3_**), 2.44 – 2.35 (m, 2H, Tyr-C^3^-C**H_2_**-), 2.33 – 2.24 (m, 4H,-NHCO-C**H_2_**-C**H_2_**-CONH-), 2.22 (s, 3H, Ar-C^4^-C**H_3_**), 1.80 – 1.68 (m, 2H, -C**H_2_**-CH_2_-O-Tyr), 1.61 – 1.50 (m, 2H, Im-NH-CH_2_-C**H_2_**-), 1.49 – 1.33 (m, 6H, -C**H_2_**-CH_2_-CH_2_-O-Tyr, Tyr-C^3^-CH_2_-C**H_2_**-CH_2_-C**H_2_**-), 1.32 – 1.18 (m, 2H, Tyr-C^3^-CH_2_-CH_2_-C**H_2_**-CH_2_-).

#### 1.3.2.17 Methyl (*R*)-3-(3-(1-azido-10,13-dioxo-3,6-dioxa-9,14-diazanonadecan-19-yl)-4-((5-((4,5 -dihydro-1H-imidazol-2-yl)amino)pentyl)oxy)phenyl)-2-((2,4,6-trimethylphenyl)sulfon amido)propanoate (14b)

Sulfonamide **10b** (404 mg, 0.54 mmol, 1.0 eq.) was dissolved in MeOH (30 mL) and Pd/C (10 %, 63.0 mg, 0.1 eq.) was added, followed by removal of the atmosphere using a waterjet pump (200 mbar) and enrichment with H_2_ (800 mbar). The resulting suspension was stirred at RT for 1 h until complete conversion determined by LCMS and filtered through celite. The solvent was removed under reduced pressure giving the crude amine (436 mg). Azido acid **13** (297 mg, 1.08 mmol, 1.9 eq.) was dissolved in dry DMF (5 mL) followed by addition of DIPEA (377 µL, 2.16 mmol, 4.0 eq.) and HATU (207 mg, 0.54 mmol, 1.0 eq.). To this yellow solution a solution of the crude amine in dry DMF (2 mL) was added and the reaction mixture stirred for 1.5 h at RT. Purification by RP-HPLC yielded conjugable RGD-mimetic precursor **14b** (291 mg, 0.33 mmol, 61 %).

M: 872.10 g/mol | Yield: 61 %, 291 mg, 0.33 mmol | C_42_H_65_N_9_O_9_S

**HPLC-MS** (ESI+):	*m*/*z* (experimental):	436.6995, t_R_ = 6.9 min.
*m*/*z* (calculated):	436.7385, [M+2H]^2+^

^1^**H-NMR** (600 MHz, methanol-*d*_4_):

δ [ppm] = 6.89 (s, 2H, Ar-C^3’3^**H**), 6.82 (dd, ^3^*J* = 8.3 Hz, ^4^*J* = 2.3 Hz, 1H, Tyr-C^6^-**H**), 6.79 (d, ^4^*J* = 2.3 Hz, 1H, Tyr-C^2^-**H**), 6.68 (d, ^3^*J* = 8.3 Hz, 1H, Tyr-C^5^-**H**), 3.95 (t, ^3^*J* = 6.1 Hz, 2H, -C**H_2_**-O-Tyr), 3.88 (dd, ^3^*J* = 9.0 Hz, ^3^*J* = 6.1 Hz, 1H, Tyr-C^α^-**H**), 3.70 (s, 4H, Im-C^3/3’^**H_2_**), 3.68 – 3.60 (m, 6H, N_3_-CH_2_-CH_2_-O-C**H_2_**-C**H_2_**-O-C**H_2_**-), 3.54 (t, ^3^*J* = 5.6 Hz, 2H, N_3_-C**H_2_**-), 3.44 (s, 3H, -C**H_3_**), 3.40 – 3.34 (m, 4H, N_3_-CH_2_-C**H_2_**-, -O-CH_2_-C**H_2_**-NHCO-), 3.23 (t, ^3^*J* = 7.1 Hz, 2H, Im-NH-C**H_2_**-), 3.15 (t, ^3^*J* = 7.1 Hz, 2H, -CH_2_-CH_2_-C**H_2_**-NHCO-), 2.89 (dd, ^2^*J* = 13.8 Hz, ^3^*J* = 6.0 Hz, 1H, C^β^-**H**/H’), 2.73 (dd, ^2^*J* = 13.8 Hz, ^3^*J* = 9.0 Hz, 1H, C^β^‑H/**H’**), 2.58 – 2.41 (m, 6H, Tyr-C^3^-C**H_2_**- ,-NHCO-C**H_2_**-C**H_2_**-CONH-), 2.48 (s, 6H, Ar-C^2/2’^-C**H_3_**), 2.27 (s, 3H, Ar-C^4^-C**H_3_**), 1.90 – 1.79 (m, 2H, -C**H_2_**-CH_2_-O-Tyr), 1.75 – 1.63 (m, 2H, Im-NH-CH_2_-C**H_2_**-), 1.62 – 1.56 (m, 2H, -C**H_2_**-CH_2_-CH_2_-O-Tyr), 1.55 – 1.47 (m, 4H, Tyr-C^3^-CH_2_-C**H_2_**-CH_2_-C**H_2_**-), 1.39 – 1.26 (m, 2H, Tyr-C^3^-CH_2_-CH_2_-C**H_2_**-CH_2_-).

^13^**C**{^1^**H**}**-NMR** (151 MHz, methanol-*d*_4_):

δ [ppm] = 174.7(-O-CH_2_-CH_2_-NH**C**O-), 174.5 (-CH_2_-CH_2_-NH**C**O-), 173.6 (-**C**OOMe), 161.3 (Im-**C^1^**), 157.1 (Tyr-**C^4^**), 143.4 (Ar-**C^4^**), 140.5 (Ar-**C^2/2’^**), 135.1 (Ar-**C^1^**), 132.8 (Ar-**C^3/3’^**), 131.9 (Tyr-**C^3^**), 131.6 (Tyr-**C^2^**), 129.2 (Tyr-**C^1^**), 128.6 (Tyr-**C^6^**), 112.1 (Tyr-**C^5^**), 71.5 (-O-**C**H_2_-**C**H_2_-O-), 71.4 (-O-**C**H_2_-**C**H_2_-O-), 71.1 (-O-**C**H_2_-CH_2_-NHCO-), 70.6 (N_3_-**C**H_2_-), 68.6 (-**C**H_2_-O-Tyr), 58.9 (Tyr-**C^α^**), 52.5 (-**C**H_3_), 51.7 (N_3_-CH_2_-**C**H_2_-), 44.1 (Im-**C^3/3’^**), 43.9 (Im-NH-**C**H_2_-), 40.5 (-**C**H_2_-NHCO-CH_2_-CH_2_-CONH-**C**H_2_-), 40.4 (‑**C**H_2_-NHCO-CH_2_-CH_2_-CONH-**C**H_2_-), 38.7 (Tyr-**C^β^**), 32.3 (-NHCO-**C**H_2_-**C**H_2_-CONH-), 32.3 (-NHCO-**C**H_2_-**C**H_2_-CONH-), 31.2 (Tyr-C^3^-**C**H_2_-), 30.7 (-CH_2_-**C**H_2_-CH_2_-NHCO-), 30.3 (Tyr-C^3^-CH_2_-**C**H_2_-), 30.2 (-**C**H_2_-CH_2_-O-Tyr), 30.0 (Im-NH-CH_2_-**C**H_2_-), 27.8 (Tyr-C^3^-CH_2_-CH_2_-**C**H_2_-), 24.6 (Im-NH-CH_2_-CH_2_-**C**H_2_-), 23.2 (Ar-C^2/2’^-**C**H_3_), 21.0 (Ar-C^4^-**C**H_3_).

#### 1.3.2.18 (*S*)-3-(3-(1-azido-10,13-dioxo-3,6-dioxa-9,14-diazanonadecan-19-yl)-4-((5-((4,5-dihydro-1H-imidazol-2-yl)amino)pentyl)oxy)phenyl)-2-((2,4,6-trimethylphenyl) sulfonamido)propanoic acid (15a)

RGD-mimetic precursor **14a** (227 mg, 0.26 mmol, 1.0 eq.) was dissolved in MeOH (1.5 mL) followed by addition of a suspension of LiOH∙H_2_O (109 mg, 2.60 mmol, 10 eq.) in H_2_O (2 mL). The reaction mixture was stirred at RT for 4 h until complete conversion determined by LCMS. The suspension was centrifuged and the supernatant purified by RP-HPLC. Freeze-drying yielded RGD-mimetic **15a** (167 mg, 0.20 mmol, 75 %) as colorless oil.

M: 858.07 g/mol | Yield: 75 %, 167 mg, 195 µmol | C_41_H_63_N_9_O_9_S

**UHPLC-MS** (ESI+):	*m*/*z* (experimental):	858.4537, t_R_ = 2.2 min.
*m*/*z* (calculated):	858.4542, [M+H]^+^

^1^**H-NMR** (500 MHz, DMSO–*d*_6_):

δ [ppm] = 8.31 (t, ^3^*J* = 5.8 Hz, 1H, Im-N**H**), 8.00 (d, ^3^*J* = 9.4 Hz, 1H, -SO_2_-N**H**-), 7.86 (t, ^3^*J* = 5.7 Hz, 1H, -O-CH_2_-CH_2_-N**H**-), 7.80 (t, ^3^*J* = 5.6 Hz, 1H, -O-CH_2_-CH_2_-NHCO-CH_2_-CH_2_-CON**H**-), 6.83 (s, 2H, Ar-C^3’3^**H**), 6.81 (dd, ^3^*J* = 8.4 Hz, ^4^*J* = 2.2 Hz, 1H, Tyr-C^6^-**H**), 6.73 (d, ^4^*J* = 2.2 Hz, 1H, Tyr-C^2^-**H**), 6.64 (d, ^3^*J* = 8.3 Hz, 1H, Tyr-C^5^-**H**), 3.88 (t, ^3^*J* = 6.3 Hz, 2H, -C**H_2_**-O-Tyr), 3.68 (dd, ^3^*J* = 9.5 Hz, ^3^*J* = 5.2 Hz, 1H, Tyr-C^α^-**H**), 3.63 – 3.57 (m, 6H, Im-C^3/3’^**H_2_**, -C**H_2_**-CH_2_-N_3_), 3.56 – 3.53 (m, 2H, -O-C**H_2_**-CH_2_‑O‑), 3.52 – 3.49 (m, 2H, -O-CH_2_-C**H_2_**‑O‑), 3.42 – 3.36 (m, 4H, N_3_-C**H_2_**-CH_2_-O-CH_2_-CH_2_-O-C**H_2_**‑), 3.22 – 3.12 (m, 4H, Im-NH-C**H_2_**-, -O-CH_2_-C**H_2_**-NHCO-), 3.01 (dd, ^3^*J* = 6.7 Hz, ^3^*J* = 6.7 Hz, 2H, -CH_2_-CH_2_-C**H_2_**-NHCO-), 2.82 (dd, ^2^*J* = 13.8 Hz, ^3^*J* = 5.1 Hz, 1H, C^β^-**H**/H’), 2.60 (dd, ^2^*J* = 13.8 Hz, ^3^*J* = 9.6 Hz, 1H, C^β^-H/**H’**), 2.40 (s, 6H, Ar-C^2/2’^-C**H_3_**), 2.41 – 2.32 (m, 2H, Tyr-C^3^-C**H_2_**-), 2.30 – 2.27 (m, 4H, -NHCO-C**H_2_**-C**H_2_**-CONH-), 2.21 (s, 3H, Ar-C^4^-C**H_3_**), 1.77 – 1.70 (m, 2H, -C**H_2_**-CH_2_-O-Tyr), 1.61 – 1.52 (m, 2H, Im-NH-CH_2_-C**H_2_**-), 1.49 – 1.35 (m, 6H, -C**H_2_**-CH_2_-CH_2_-O-Tyr, Tyr-C^3^-CH_2_-C**H_2_**-CH_2_-C**H_2_**-), 1.30 – 1.21 (m, 2H, Tyr-C^3^-CH_2_-CH_2_-C**H_2_**-CH_2_-).

^13^**C**{^1^**H**}**-NMR** (151 MHz, DMSO–*d*_6_):

δ [ppm] = 172.8 (-**C**OOH), 171.5 (-O-CH_2_-CH_2_-NH**C**O-), 171.2 (-CH_2_-CH_2_-CH_2_-NH**C**O-), 159.4 (Im‑**C^1^**), 155.0 (Tyr-**C^4^**), 140.9 (Ar-**C^4^**), 138.1 (Ar-**C^2/2’^**), 134.5 (Ar-**C^1^**), 131.3 (Ar-**C^3/3’^**), 129.8 (Tyr-**C^3^**), 129.7 (Tyr-**C^2^**), 128.2 (Tyr-**C^1^**), 127.3 (Tyr-**C^6^**), 110.8 (Tyr-**C^5^**), 69.6 (-O-**C**H_2_-**C**H_2_-O-), 69.6 (-O-**C**H_2_-**C**H_2_-O-), 69.3 (N_3_-CH_2_-**C**H_2_-), 69.1 (-O-**C**H_2_-CH_2_-NHCO-), 67.1 (-**C**H_2_-O-Tyr), 57.3 (Tyr-**C^α^**), 50.0 (N_3_-**C**H_2_-), 42.5 (Im-**C^3/3’^**), 42.2 (Im-NH-**C**H_2_-), 38.5 (-**C**H_2_-NHCO-CH_2_-CH_2_-CONH-**C**H_2_-), 38.5 (-**C**H_2_-NHCO-CH_2_-CH_2_-CONH-**C**H_2_-), 36.9 (Tyr-**C^β^**), 30.8 (-NHCO-**C**H_2_-**C**H_2_-CONH-), 30.7 (-NHCO-**C**H_2_-**C**H_2_-CONH-), 29.6 (Tyr-C^3^-**C**H_2_-), 29.1 (Tyr-C^3^-CH_2_-**C**H_2_-CH_2_-**C**H_2_-), 29.0 (Tyr-C^3^-CH_2_-**C**H_2_-CH_2_-**C**H_2_-), 28.5 (Im-NH-CH_2_-**C**H_2_-CH_2_-**C**H_2_-), 28.4 (Im-NH-CH_2_-**C**H_2_-CH_2_-**C**H_2_-), 26.5 (Tyr-C^3^-CH_2_-CH_2_-**C**H_2_-), 22.8 (Im-NH-CH_2_-CH_2_-**C**H_2_-), 22.6 (Ar-C^2/2’^-**C**H_3_), 20.4 (Ar-C^4^-**C**H_3_).

#### 1.3.2.19 (*R*)-3-(3-(1-azido-10,13-dioxo-3,6-dioxa-9,14-diazanonadecan-19-yl)-4-((5-((4,5-dihydro-1H-imidazol-2-yl)amino)pentyl)oxy)phenyl)-2-((2,4,6-trimethylphenyl) sulfonamido)propanoic acid (15b)

RGD-mimetic precursor **14b** (291 mg, 0.33 mmol, 1.0 eq.) was dissolved in MeOH (1.5 mL) followed by addition of a suspension of LiOH∙H_2_O (140 mg, 3.33 mmol, 10 eq.) in H_2_O (2 mL). The reaction mixture was stirred at RT for 3 h until complete conversion determined by LCMS. The suspension was centrifuged and the supernatant purified by RP-HPLC. Freeze-drying yielded RGD-mimetic **15b** (168 mg, 0.20 mmol, 59 %) as colorless oil.

M: 858.07 g/mol | Yield: 59 %, 168 mg, 196 µmol | C_41_H_63_N_9_O_9_S

**UHPLC-MS** (ESI+):	*m*/*z* (experimental):	858.4239, t_R_ = 2.3 min.
*m*/*z* (calculated):	858.4542, [M+H]^+^

^1^**H-NMR** (600 MHz, methanol-*d*_4_):

δ [ppm] = 6.86 (s, 2H, Ar-C^3’3^**H**), 6.83 (dd, ^3^*J* = 8.3 Hz, ^4^*J* = 2.5 Hz, 1H, Tyr-C^6^-**H**), 6.78 (d, ^4^*J* = 2.5 Hz, 1H, Tyr-C^2^-**H**), 6.65 (d, ^3^*J* = 8.4 Hz, 1H, Tyr-C^5^-**H**), 3.94 (t, ^3^*J* = 6.1 Hz, 2H, -C**H_2_**-O-Tyr), 3.85 (dd, ^3^*J* = 9.2 Hz, ^3^*J* = 5.1 Hz, 1H, Tyr-C^α^-**H**), 3.69 (s, 4H, Im-C^3/3’^**H_2_**), 3.68 – 3.59 (m, 6H, N_3_-CH_2_-CH_2_-O-C**H_2_**-C**H_2_**-O-C**H_2_**-), 3.54 (t, ^3^*J* = 5.6 Hz, 2H, N_3_-C**H_2_**-), 3.42 – 3.33 (m, 4H, N_3_-CH_2_-C**H_2_**-, -O-CH_2_-C**H_2_**-NHCO-), 3.24 (t, ^3^*J* = 7.1 Hz, 2H, Im-NH-C**H_2_**-), 3.15 (t, ^3^*J* = 7.1 Hz, 2H, -CH_2_-CH_2_-C**H_2_**-NHCO-), 2.94 (dd, ^2^*J* = 13.9 Hz, ^3^*J* = 5.2 Hz, 1H, C^β^-**H**/H’), 2.71 (dd, ^2^*J* = 13.9 Hz, ^3^*J* = 9.2 Hz, 1H, C^β^-H/**H’**), 2.60 – 2.37 (m, 6H, Tyr-C^3^-C**H_2_**- ,-NHCO-C**H_2_**-C**H_2_**-CONH-), 2.46 (s, 6H, Ar-C^2/2’^-C**H_3_**), 2.26 (s, 3H, Ar-C^4^-C**H_3_**), 1.88 – 1.80 (m, 2H, -C**H_2_**-CH_2_-O-Tyr), 1.73 – 1.65 (m, 2H, Im-NH-CH_2_-C**H_2_**-), 1.61 – 1.55 (m, 2H, -C**H_2_**-CH_2_-CH_2_-O-Tyr), 1.56 – 1.46 (m, 4H, Tyr-C^3^-CH_2_-C**H_2_**-CH_2_-C**H_2_**-), 1.37 – 1.28 (m, 2H, Tyr-C^3^-CH_2_-CH_2_-C**H_2_**-CH_2_-).

^13^**C**{^1^**H**}**-NMR** (151 MHz, methanol-*d*_4_):

δ [ppm] = 174.9 (-**C**OOH), 174.7 (-O-CH_2_-CH_2_-NH**C**O-), 174.5 (-CH_2_-CH_2_-NH**C**O-), 161.3 (Im-**C^1^**), 157.0 (Tyr-**C^4^**), 143.2 (Ar-**C^4^**), 140.3 (Ar-**C^2/2’^**), 135.3 (Ar-**C^1^**), 132.8 (Ar-**C^3/3’^**), 131.7 (Tyr-**C^3^**), 131.6 (Tyr-**C^2^**), 129.5 (Tyr-**C^1^**), 128.7 (Tyr-**C^6^**), 112.0 (Tyr-**C^5^**), 71.5 (-O-**C**H_2_-**C**H_2_-O-), 71.4 (-O-**C**H_2_-**C**H_2_-O-), 71.1 (-O-**C**H_2_-CH_2_-NHCO-), 70.6 (N_3_-**C**H_2_-), 68.6 (-**C**H_2_-O-Tyr), 58.8 (Tyr-**C^α^**), 51.7 (N_3_-CH_2_-**C**H_2_-), 44.0 (Im-**C^3/3’^**), 43.9 (Im-NH-**C**H_2_-), 40.5 (-**C**H_2_-NHCO-CH_2_-CH_2_-CONH-**C**H_2_-), 40.4 (‑**C**H_2_-NHCO-CH_2_-CH_2_-CONH-**C**H_2_-), 38.9 (Tyr-**C^β^**), 32.3 (-NHCO-**C**H_2_-**C**H_2_-CONH-), 32.3 (-NHCO-**C**H_2_-**C**H_2_-CONH-), 31.3 (Tyr-C^3^-**C**H_2_-), 30.7 (-CH_2_-**C**H_2_-CH_2_-NHCO-), 30.3 (Tyr-C^3^-CH_2_-**C**H_2_-), 30.2 (-**C**H_2_-CH_2_-O-Tyr), 30.0 (Im-NH-CH_2_-**C**H_2_-), 27.8 (Tyr-C^3^-CH_2_-CH_2_-**C**H_2_-), 24.6 (Im-NH-CH_2_-CH_2_-**C**H_2_-), 23.2 (Ar-C^2/2’^-**C**H_3_), 21.1 (Ar-C^4^-**C**H_3_).

#### 1.3.2.20 Methyl (*S*)-2-amino-3-(4-hydroxy-3-iodophenyl)propanoate (S1a)

A mixture of iodo-l-tyrosine **2a** (11.2 g, 36.6 mmol, 1.0 eq.), diiodo-l-tyrosine (639 mg, 1.47 mmol) and l‑tyrosine (895 mg, 4.94 mmol) was dissolved in methanol (100 mL) and thionyl chloride (6.20 mL, 85.2 mmol, 2.3 eq.) was added dropwise while cooling with an ice bath. Afterwards the orange solution was heated to reflux for 1 h. Removing the solvent under reduced pressure and further drying in HV yielded a crude (14.8 g) containing methyl ester **S1a**, diiodo-l‑tyrosine methyl ester and l-tyrosine methyl ester in a molar ratio of 90:2:8. The crude was used in the next step without further purification.

M: 321.11 g/mol | C_10_H_12_INO_3_

^1^**H-NMR** (500 MHz, methanol-*d*_4_):

δ [ppm] = 7.62 (d, ^4^*J* = 2.2 Hz, 1H, C^2^-**H**), 7.09 (dd, ^3^*J* = 8.3 Hz, ^4^*J* = 2.3 Hz, 1H, C^6^-**H**), 6.84 (d, ^3^*J* = 8.3 Hz, 1H, C^5^-**H**), 4.27 (dd, ^3^*J* = 7.5 Hz, ^3^*J* = 6.0 Hz, 1H, C^α^-**H**), 3.84 (s, 3H, -OC**H_3_**), 3.16 (dd, ^2^*J* = 14.6, ^3^*J* = 6.0 Hz, 1H, C^β^-**H**/H’), 3.06 (dd, ^2^*J* = 14.6, ^3^*J* = 7.5 Hz, 1H, C^β^-H/**H’**).

#### 1.3.2.21 Methyl (*R*)-2-amino-3-(4-hydroxy-3-iodophenyl)propanoate (S1b)

A mixture of iodo-d-tyrosine **2b** (15.6 g, 50.7 mmol, 1.0 eq.), diiodo-d-tyrosine (2.42 g, 5.58 mmol) and d‑tyrosine (874 mg, 4.82 mmol) was dissolved in methanol (100 mL) and thionyl chloride (7.50 mL, 103 mmol, 2.0 eq.) was added dropwise while cooling with an ice bath. Afterwards the orange solution was heated to reflux for 1 h. Removing the solvent under reduced pressure and further drying in HV yielded a crude (19.7 g) containing methyl ester **S1b** (14.4 g, 44.8 mmol, 80 %) in a mixture with diiodo-d-tyrosine methyl ester (1.65 g, 3.69 mmol, 7 %), d-tyrosine methyl ester (0.96 g, 4.93 mmol, 9 %) and residual methanol (3.00 g). The crude was used in the next step without further purification.

M: 321.11 g/mol | Yield: 80 %, 14.4 g, 44.8 mmol | C_10_H_12_INO_3_

^1^**H-NMR** (500 MHz, methanol-*d*_4_):

δ [ppm] = 7.60 (s, 1H, C^2^-**H**), 7.09 (d, ^3^*J* = 8.4 Hz, 1H, C^6^-**H**), 6.83 (d, ^3^*J* = 8.2 Hz, 1H, C^5^-**H**), 4.25 (t, ^3^*J* = 6.8 Hz, ^3^*J* = 6.8 Hz, 1H, C^α^-**H**), 3.81 (s, 3H, -OC**H_3_**), 3.14 (dd, ^2^*J* = 14.4 Hz, ^3^*J* = 6.0 Hz, 1H, C^β^-**H**/H’), 3.06 (dd, ^2^*J* = 14.7 Hz, ^3^*J* = 7.5 Hz, 1H, C^β^-H/**H’**).

#### 1.3.2.22 (Ethane-1,2-diylbis(oxy))bis(ethane-2,1-diyl)bis(4-methylbenzenesulfonate) (S2)

Triethylene glycol **(11)** (7.52 g, 50.1 mmol, 1.0 eq.) was dissolved in DCM (50 mL) and TosylCl (19.1 g, 100 mmol, 2.0 eq.) was added. KOH (22.4 g, 399 mmol, 7.9 eq.) was added portion wise while cooling with an ice bath. The reaction mixture was stirred at 0 °C for 3.5 h until complete conversion determined by TLC. DCM (100 mL) and water (100 mL) were added and the phases separated. The aqueous phase was extracted with DCM (2x50 mL) and the combined organic layers washed with brine (2x75 mL), as well as dried over MgSO_4_. Filtration and removing the solvent under reduced pressure yielded ditosylate **S2** (21.5 g, 46.7 mmol, 95 %) as colorless solid.

M: 458.54 g/mol | Yield: 95 %, 21.5 g, 46.7 mmol | C_20_H_26_O_8_S_2_

**DC**: R*_f_* (dichloromethane/methanol, 95:5) = 0.80.

**UHPLC-MS** (ESI+):	*m*/*z* (experimental):	459.2063, t_R_ = 3.0 min.
*m*/*z* (calculated):	459.1142, [M+H]^+^

^1^**H-NMR** (500 MHz, CDCl_3_):

δ [ppm] = 7.78 (d, ^3^*J* = 7.5 Hz, 4H, Ar-C^2/2’^**H**), 7.34 (d, ^3^*J* = 7.8 Hz, 4H, Ar-C^3/3’^**H**), 4.22 – 4.04 (m, 4H, -SOC**H_2_**-), 3.77 – 3.60 (m, 4H, -SOCH_2_-C**H_2_**-), 3.52 (s, 4H, -SOCH_2_-CH_2_-O-C**H_2_**-), 2.44 (s, 6H, -C**H_3_**).

^13^**C**{^1^**H**}**-NMR** (126 MHz, CDCl_3_):

δ [ppm] = 145.0 (Ar-**C^1^**), 133.1 (Ar-**C^4^**), 130.0 (Ar-**C^2/2‘^**), 128.1 (Ar-**C^3/3‘^**), 70.8 (-SO**C**H_2_-), 69.3 (‑SOCH_2_-**C**H_2_-), 68.9 (-SOCH_2_-CH_2_-O-**C**H_2_-), 21.8 (-**C**H_3_).

#### 1.3.2.23 1,2-bis(2-azidoethoxy)ethane (S3)

Ditosylate **S2** (21.5 g, 46.7 mmol, 1.0 eq.), NaN_3_ (12.4 g, 191 mmol, 4.1 eq.) and TBAI (879 mg, 2.38 mmol, 0.05 eq.) were suspended in DMF (100 mL) and stirred at 80 °C for 22 h until complete conversion determined by LCMS. Afterwards the solvent was removed under reduced pressure and diethyl ether (100 mL) added to the crude. Filtration of insoluable material and removing the solvent under reduced pressure yielded azide **S3** (9.35 g, 46.7 mmol, quant.) as yellow liquid.

M: 200.20 g/mol | Yield: quant., 9.35 g, 46.7 mmol | C_6_H_12_N_6_O_2_

^1^**H-NMR** (500 MHz, CDCl_3_):

δ [ppm] = 3.74 – 3.61 (m, 8H, N_3_-C**H_2_**-C**H_2_**-), 3.39 (s, 4H, N_3_-CH_2_-CH_2_-O-C**H_2_**-).

### 1.3.3 Linker

#### 1.3.3.1 Pent-4-ynoyl-l-asparaginyl-l-prolyl-l-valine (16a)

2-Chlorotrityl chloride resin (1.60 mmol∙g^-1^, 1.02 g, 1.63 mmol, 1.0 eq.) was swollen in dry DCM (20 mL) for 30 minutes, filtered and washed with dry DCM (2x15 mL). Fmoc-Val-OH (1.34 g, 3.95 mmol, 2.4 eq.) in dry DCM (12 mL) and DIPEA (3.00 mL, 17.6 mmol, 11 eq.) was added and the syringe was shaken for 21 h at RT. To deactivate remaining binding sites MeOH (2 mL) was added and the syringe was shaken for further 2 h at RT. Then the suspension was filtered and the resin washed with DCM (5x5 mL), DMF (5x5 mL), DCM (5x5 mL) and Et_2_O (5x5 mL) and dried in vacuum to yield dry resin beads. The loading was determined after *GP I* to 1.00 mmol∙g^-1^. The loaded resin was swollen in DMF (10 mL) for 15 minutes and the suspension filtered. The reason was deprotected after *GP II* and then coupled with Fmoc-Pro-OH after *GP III*. These steps were repeated consecutively with Fmoc-Asn(Trt)-OH and 4-pentynoic acid. After every coupling step *GP IV* was performed to determine successful coupling. After completion of all coupling steps the resin was swollen in DCM (10 mL) and a solution of TFA/TIS/H_2_O (95:2.5:2.5, v:v:v, 10% in DCM, 5x2 mL) was added and incubated for 5x2 minutes at RT. The suspension was filtered each time and the combined filtrate concentrated in vacuum. Purification of the resulting crude by RP-HPLC and freeze-drying yielded Pentynoyl-Asn-Pro-Val-OH **16a** (377 mg, 0.92 mmol, 91 %) as colorless solid.

M: 408.46 g/mol | Yield: 91 %, 377 mg, 0.92 mmol | C_19_H_28_N_4_O_6_

**HPLC-MS** (ESI+):	*m*/*z* (experimental):	409.1823, t_R_ = 4.8 min.
*m*/*z* (calculated):	409.2082, [M+H]^+^

**HRMS** (ESI+):	*m*/*z* (experimental):	431.1893,
*m*/*z* (calculated):	431.1901, [M+Na]^+^

Rotamers were observed in the molar ratio of 90:10.

^1^**H-NMR** (600 MHz, DMSO–*d*_6_):

δ [ppm] = 8.22 (d, ^3^*J* = 7.8 Hz, 0.9H, Asn-N**H**, major rot.), 8.01 (d, ^3^*J* = 7.6 Hz, 0.1H, Val-N**H**, minor rot.), 7.90 (d, ^3^*J* = 8.5 Hz, 1H, Val-N**H** major rot., Asn-N**H** minor rot.), 7.34 (br s, 0.9H, Asn-N**H**/H‘, major rot.), 7.30 (br s, 0.1H, Asn-N**H**/H‘, minor rot.), 6.86 (br s, 0.9H, Asn-NH/**H‘**, major rot.), 6.79 (br s, 0.1H, Asn-NH/**H‘**, minor rot.), 4.82 (dd, ^3^*J* = 7.2 Hz, ^3^*J* = 7.2 Hz, 0.9H, Asn-C^α^**H**, major rot.), 4.77 (dd, ^3^*J* = 8.1 Hz, ^3^*J* = 2.1 Hz, 0.1H, Pro-C^α^**H**, minor rot.), 4.73 – 4.67 (m, 0.1H, Asn-C^α^**H**, minor rot.), 4.41 (dd, ^3^*J* = 8.3 Hz, ^3^*J* = 2.6 Hz, 0.9H, Pro-C^α^**H**, major rot.), 4.04 (dd, ^3^*J* = 8.5 Hz, ^3^*J* = 6.1 Hz, 0.9H, Val-C^α^**H**, major rot.), 3.95 (dd, ^3^*J* = 7.6 Hz, ^3^*J* = 5.6 Hz, 0.1H, Val-C^α^**H**, minor rot.), 3.74 – 3.57 (m, 1.8H, Pro-C^5^**H_2_**, major rot.), 3.39 – 3.34 (m, 0.2H, Pro-C^5^**H_2_**, minor rot.), 2.74 (t, ^4^*J* = 2.5 Hz, 0.9H, ‑C≡C**H**, major rot.), 2.73 (t, ^4^*J* = 2.4 Hz, 0.1H, ‑C≡C**H**, minor rot.), 2.55 – 2.51 (m, 1H, Asn-C^β^**H**/H‘), 2.36 – 2.25 (m, 5H, Asn-C^β^H/**H‘**, -C**H_2_**-C**H_2_**-C≡CH), 2.04 (dq, ^3^*J* = 13.5 Hz, ^3^*J* = 6.7 Hz, 1H, Val-C^β^**H**), 2.00 – 1.94 (m, 1H, Pro-C^β^**H**/H‘), 1.90 – 1.82 (m, 3H, Pro-C^β^H/**H‘**, Pro-C^4^**H_2_**), 0.92 (d, ^3^*J* = 6.9 Hz, 0.3H, Val-C^β^(C**H_3_**)^A^(CH_3_)^B^, minor rot.), 0.89 (d, ^3^*J* = 7.0 Hz, 0.3H, Val-C^β^(CH_3_)^A^(C**H_3_**)^B^, minor rot.), 0.87 (d, ^3^*J* = 3.3 Hz, 2.7H, Val-C^β^(C**H_3_**)^A^(CH_3_)^B^, major rot.), 0.86 (d, ^3^*J* = 3.3 Hz, 2.7H, Val‑C^β^(CH_3_)^A^(C**H_3_**)^B^, major rot.).

^13^**C**{^1^**H**}**-NMR** (151 MHz, DMSO–*d*_6_):

δ [ppm] = 172.8 (-**C**OOH), 171.4 (Pro-**C**=O), 171.2 (Asn-**C**ONH_2_), 170.0 (Asn-**C**=O), 169.9 (-CH_2_-**C**ONH-), 83.7(‑**C**≡CH), 71.3 (‑C≡**C**H), 59.2 (Pro-**C^α^**H), 57.3 (Val-**C^α^**H), 47.5 (Asn-**C^α^**H), 46.7 (Pro-**C^5^**H_2_), 37.3 (Asn-**C^β^**H_2_), 33.7 (HNCO-**C**H_2_-), 29.7 (Val-**C^β^**H), 28.9 (Pro-**C^β^**H_2_), 24.2 (Pro-**C^4^**H_2_), 19.2 (Val-C^β^(**C**H_3_)^A^(CH_3_)^B^), 18.2 (Val-C^β^(CH_3_)^A^(**C**H_3_)^B^), 14.1 (-**C**H_2_-C≡CH).

#### 1.3.3.2 Pent-4-ynoyl-l-asparaginyl-l-prolyl-d-valine (16b)

2-Chlorotrityl chloride resin (1.60 mmol∙g^-1^, 1.00 g, 1.60 mmol, 1.0 eq.) was swollen in dry DCM (20 mL) for 30 minutes, filtered and washed with dry DCM (2x15 mL). Fmoc-d-Val-OH (1.36 g, 4.01 mmol, 2.5 eq.) in dry DCM (12 mL) and DIPEA (3.00 mL, 17.6 mmol, 11 eq.) was added and the syringe was shaken for 20 h at RT. To deactivate remaining binding sites MeOH (2 mL) was added and the syringe was shaken for further 2 h at RT. Then the suspension was filtered and the resin washed with DCM (5x5 mL), DMF (5x5 mL), DCM (5x5 mL) and Et_2_O (5x5 mL) and dried in vacuum to yield dry resin beads. The loading was determined after *GP I* to 0.90 mmol∙g^-1^. The loaded resin was swollen in DMF (10 mL) for 15 minutes and the suspension filtered. The reason was deprotected after *GP II* and then coupled with Fmoc-Pro-OH after *GP III*. These steps were repeated consecutively with Fmoc-Asn(Trt)-OH and 4-pentynoic acid. After every coupling step *GP IV* was performed to determine successful coupling. After completion of all coupling steps the resin was swollen in DCM (10 mL) and a solution of TFA/TIS/H_2_O (95:2.5:2.5, v:v:v, 10% in DCM, 5x2 mL) was added and incubated for 5x2 minutes at RT. The suspension was filtered each time and the combined filtrate concentrated in vacuum. Purification of the resulting crude by RP-HPLC and freeze-drying yielded Pentynoyl-Asn-Pro-d-Val-OH **16b** (377 mg, 0.92 mmol, 80 %) as colorless solid.

M: 408.46 g/mol | Yield: 80 %, 377 mg, 0.92 mmol | C_19_H_28_N_4_O_6_

**HPLC-MS** (ESI+):	*m*/*z* (experimental):	409.1902, t_R_ = 5.2 min.
*m*/*z* (calculated):	409.2082, [M+H]^+^

**HRMS** (ESI+):	*m*/*z* (experimental):	431.1892,
*m*/*z* (calculated):	431.1901, [M+Na]^+^

Rotamers were observed in the molar ratio of 95:05.

^1^**H-NMR** (600 MHz, DMSO–*d*_6_):

δ [ppm] = 8.27 (d, ^3^*J* = 8.1 Hz, 1H, Asn-N**H**, major rot.), 8.00 – 7.91 (m, 0.1H, Val-N**H**, minor rot., Asn-N**H** minor rot.), 7.63 (d, ^3^*J* = 8.4 Hz, 1H, Val-N**H** major rot.), 7.46 (br s, 1H, Asn-N**H**/H‘, major rot.), 7.32 (br s, 0.05H, Asn-N**H**/H‘, minor rot.), 6.96 (br s, 1H, Asn-NH/**H‘**, major rot.), 6.83 (br s, 0.05H, Asn-NH/**H‘**, minor rot.), 4.81 (td, ^3^*J* = 8.4 Hz, ^3^*J* = 5.8 Hz, 1H, Asn-C^α^**H**, major rot.), 4.75 (dd, ^3^*J* = 7.6 Hz, ^3^*J* = 2.4 Hz, 0.05H, Pro-C^α^**H**, minor rot.), 4.65 (td, ^3^*J* = 8.3 Hz, ^3^*J* = 5.5 Hz, 0.05H, Asn-C^α^**H**, minor rot.), 4.31 (dd, ^3^*J* = 8.3 Hz, ^3^*J* = 2.4 Hz, 1H, Pro-C^α^**H**, major rot.), 4.14 (dd, ^3^*J* = 8.4 Hz, ^3^*J* = 5.7 Hz, 0.05H, Val-C^α^**H**, minor rot.), 4.02 (dd, ^3^*J* = 8.4 Hz, ^3^*J* = 7.1 Hz, 1H, Val-C^α^**H**, major rot.), 3.72 (t, ^3^*J* = 6.6 Hz, 2H, Pro-C^5^**H_2_**, major rot.), 3.47 – 3.28 (m, 0.1H, Pro-C^5^**H_2_**, minor rot.), 2.74 (t, ^4^*J* = 2.5 Hz, 1H, ‑C≡C**H**, major rot.), 2.72 (t, ^4^*J* = 2.7 Hz, 0.05H, ‑C≡C**H**, minor rot.), 2.65 (dd, ^2^*J* = 15.6 Hz, ^3^*J* = 8.7 Hz, 1H, Asn-C^β^**H**/H‘, major rot.), 2.56 (dd, ^2^*J* = 15.2 Hz, ^3^*J* = 8.9 Hz, 0.05H, Asn-C^β^**H**/H‘, minor rot.), 2.44 – 2.21 (m, 5H, Asn-C^β^H/**H‘**, -C**H_2_**-C**H_2_**-C≡CH), 2.12 – 1.93 (m, 2H, Val-C^β^**H**, Pro-C^β^**H**/H‘), 1.94 – 1.78 (m, 3H, Pro-C^β^H/**H‘**, Pro-C^4^**H_2_**), 0.85 (d, ^3^*J* = 6.8 Hz, 3H, Val-C^β^(C**H_3_**)^A^(CH_3_)^B^), 0.80 (d, ^3^*J* = 6.7 Hz, 3H, Val-C^β^(CH_3_)^A^(C**H_3_**)^B^).

^13^**C**{^1^**H**}**-NMR** (151 MHz, DMSO–*d*_6_):

δ [ppm] = 172.9 (-**C**OOH), 171.8 (Asn-**C**ONH_2_), 171.2 (Pro-**C**=O), 170.2 (Asn-**C**=O), 169.9 (-CH_2_-**C**ONH-), 83.7(‑**C**≡CH), 71.3 (‑C≡**C**H), 59.8 (Pro-**C^α^**H), 57.6 (Val-**C^α^**H), 47.1 (Asn-**C^α^**H), 46.8 (Pro-**C^5^**H_2_), 37.5 (Asn-**C^β^**H_2_), 33.7 (HNCO-**C**H_2_-), 29.7 (Val-**C^β^**H), 29.4 (Pro-**C^β^**H_2_), 23.8 (Pro-**C^4^**H_2_), 19.2 (Val-C^β^(**C**H_3_)^A^(CH_3_)^B^), 18.5 (Val-C^β^(CH_3_)^A^(**C**H_3_)^B^), 14.1 (-**C**H_2_-C≡CH).

#### 1.3.3.3 (*S*)-N-((*S*)-1-((4-(hydroxymethyl)phenyl)amino)-3-methyl-1-oxobutan-2-yl)-1-(pent-4-ynoyl-l-asparaginyl)pyrrolidine-2-carboxamide (17a)

Pentynoyl-Asn-Pro-Val-OH **16a** (100 mg, 245 µmol, 1.0 eq.) was dissolved in a mixture of DCM/MeOH (2:1, v:v, 6 mL) and EEDQ (139 mg, 563 µmol, 2.3 eq.) and para-Aminobenzyl alcohol (69.0 mg, 563 µmol, 2.3 eq.) were added. The resulting solution was stirred in a closed vial for 24 h at 40 °C until complete conversion determined by LCMS. The solvent was removed under reduced pressure and the crude purified by RP-HPLC. Freezy drying yielded Pentynoyl-Asn-Pro-Val-PAB-OH **17a** (53.0 mg, 103 µmol, 42 %) as pale-yellow solid.

M: 513.60 g/mol | Yield: 42 %, 53.0 mg, 103 µmol | C_26_H_35_N_5_O_6_

**HPLC-MS** (ESI+):	*m*/*z* (experimental):	514.2201, t_R_ = 5.6 min.
*m*/*z* (calculated):	514.2660, [M+H]^+^

**HRMS** (ESI+):	*m*/*z* (experimental):	536.2480,
*m*/*z* (calculated):	536.2479, [M+Na]^+^

^1^**H-NMR** (600 MHz, DMSO–d_6_):

δ [ppm] = 9.41 (s, 1H, Ar-C^1^-N**H**-), 8.34 (d, ^3^*J* = 7.9 Hz, 1H, Asn-N**H**), 7.84 (d, ^3^*J* = 8.8 Hz, 1H, Val‑NH), 7.58 (d, ^3^*J* = 8.5 Hz, 2H, Ar-C^2/2‘^**H**), 7.50 (br s, 1H, Asn-N**H**/H‘), 7.23 (d, ^3^*J* = 8.3 Hz, 2H, Ar‑C^3/3‘^**H**), 6.98 (br s, 1H, Asn-NH/**H‘**), 4.85 (td, ^3^*J* = 8.1 Hz, ^3^*J* = 5.9 Hz, 1H, Asn-C^α^**H**), 4.43 (s, 2H, Ar-C^4^-C**H_2_**-), 4.36 (dd, ^3^*J* = 8.5 Hz, ^3^*J* = 3.1 Hz, 1H, Pro-C^α^**H**), 4.18 (dd, ^3^*J* = 8.7 Hz, ^3^*J* = 7.1 Hz, 1H, Val-C^α^**H**), 3.84 – 3.74 (m, 1H, Pro-C^5^**H**/H‘), 3.73 – 3.65 (m, 1H, Pro-C^5^H/**H‘**), 2.76 (t, ^4^*J* = 2.4 Hz, 1H, ‑C≡C**H**), 2.65 (dd, ^2^*J* = 15.4 Hz, ^3^*J* = 8.4 Hz, 1H, Asn-C^β^**H**/H‘), 2.41 – 2.26 (m, 5H, Asn-C^β^H/**H‘**, ‑C**H_2_**‑C**H_2_**-C≡CH), 2.20 – 2.10 (m, 1H, Val-C^β^**H**), 2.10 – 2.01 (m, 1H, Pro-C^β^**H**/H‘), 1.96 – 1.81 (m, 3H, Pro-C^β^H/**H‘**, Pro-C^4^**H_2_**), 0.91 (d, ^3^*J* = 5.2 Hz, 3H, Val-C^β^(C**H_3_**)^A^(CH_3_)^B^), 0.90 (d, ^3^*J* = 5.3 Hz, 3H, Val-C^β^(CH_3_)^A^(C**H_3_**)^B^).

^13^**C**{^1^**H**}**-NMR** (151 MHz, DMSO–d_6_):

δ [ppm] = 172.3 (Ar-C^1^-NH**C**O-), 171.9 (Asn-**C**ONH_2_), 171.2 (Pro-**C**=O), 170.5 (Asn-**C**=O), 170.1 (‑CH_2_-**C**ONH-), 138.0 (Ar‑**C^1^**), 137.8 (Ar‑**C^4^**), 127.3 (Ar‑**C^3/3‘^**), 119.5 (Ar‑**C^2/2‘^**), 84.1 (‑**C**≡CH), 71.8 (‑C≡**C**H), 63.1 (Ar-C^4^-**C**H_2_-), 60.5 (Pro-**C^α^**H), 59.4 (Val-**C^α^**H), 47.8 (Asn-**C^α^**H), 47.5 (Pro-**C^5^**H_2_), 37.9 (Asn-**C^β^**H_2_), 34.2 (HNCO-**C**H_2_-), 30.3 (Val-**C^β^**H), 29.8 (Pro-**C^β^**H_2_), 24.7 (Pro-**C^4^**H_2_), 19.9 (Val‑C^β^(**C**H_3_)^A^(CH_3_)^B^), 19.1 (Val-C^β^(CH_3_)^A^(**C**H_3_)^B^), 14.6 (-**C**H_2_-C≡CH).

#### 1.3.3.4 (S)-N-((R)-1-((4-(hydroxymethyl)phenyl)amino)-3-methyl-1-oxobutan-2-yl)-1-(pent-4-ynoyl-l-asparaginyl)pyrrolidine-2-carboxamide (17b)

Pentynoyl-Asn-Pro-d-Val-OH **16b** (100 mg, 245 µmol, 1.0 eq.) was dissolved in a mixture of DCM/MeOH (2:1, v:v, 6 mL) and EEDQ (139 mg, 563 µmol, 2.3 eq.) and para-Aminobenzyl alcohol (69.0 mg, 563 µmol, 2.3 eq.) were added. The resulting solution was stirred in a closed vial for 24 h at 40 °C until complete conversion determined by LCMS. The solvent was removed under reduced pressure and the crude purified by RP-HPLC. Freezy drying yielded Pentynoyl-Asn-Pro-d-Val-PAB-OH **17b** (74.0 mg, 144 µmol, 59 %) as pale-yellow solid.

M: 513.60 g/mol | Yield: 59 %, 74.0 mg, 144 µmol | C_26_H_35_N_5_O_6_

**HPLC-MS** (ESI+):	*m*/*z* (experimental):	514.2154, t_R_ = 5.8 min.
*m*/*z* (calculated):	514.2660, [M+H]^+^

**HRMS** (ESI+):	*m*/*z* (experimental):	536.2482,
*m*/*z* (calculated):	536.2479, [M+Na]^+^

^1^**H-NMR** (600 MHz, DMSO–d_6_):

δ [ppm] = 9.83 (s, 1H, Ar-C^1^-N**H**-), 8.29 (d, ^3^*J* = 7.9 Hz, 1H, Asn-N**H**), 7.81 (d, ^3^*J* = 8.7 Hz, 1H, Val‑NH), 7.59 (d, ^3^*J* = 8.5 Hz, 2H, Ar-C^2/2‘^**H**), 7.45 (br s, 1H, Asn-N**H**/H‘), 7.24 (d, ^3^*J* = 8.3 Hz, 2H, Ar‑C^3/3‘^**H**), 7.00 (br s, 1H, Asn-NH/**H‘**), 4.84 (td, ^3^*J* = 7.9 Hz, ^3^*J* = 6.3 Hz, 1H, Asn-C^α^**H**), 4.43 (s, 2H, Ar-C^4^-C**H_2_**-), 4.35 (dd, ^3^*J* = 8.5 Hz, ^3^*J* = 3.4 Hz, 1H, Pro-C^α^**H**), 4.18 (t, ^3^*J* = 8.2 Hz, 1H, Val-C^α^**H**), 3.78 – 3.67 (m, 2H, Pro-C^5^**H_2_**), 2.75 (t, ^4^*J* = 2.4 Hz, 1H, ‑C≡C**H**), 2.64 (dd, ^2^*J* = 15.7 Hz, ^3^*J* = 8.0 Hz, 1H, Asn-C^β^**H**/H‘), 2.39 – 2.26 (m, 5H, Asn-C^β^H/**H‘**, ‑C**H_2_**‑C**H_2_**-C≡CH), 2.14 – 2.05 (m, 1H, Val-C^β^**H**), 2.05 – 1.99 (m, 1H, Pro-C^β^**H**/H‘), 1.92 – 1.80 (m, 3H, Pro-C^β^H/**H‘**, Pro-C^4^**H_2_**), 0.87 (d, ^3^*J* = 6.7 Hz, 3H, Val-C^β^(C**H_3_**)^A^(CH_3_)^B^), 0.84 (d, ^3^*J* = 6.6 Hz, 3H, Val-C^β^(CH_3_)^A^(C**H_3_**)^B^).

^13^**C**{^1^**H**}**-NMR** (151 MHz, DMSO–d_6_):

δ [ppm] = 171.7 (Ar-C^1^-NH**C**O-), 171.4 (Asn-**C**ONH_2_), 170.2 (Pro-**C**=O), 170.0 (Asn-**C**=O), 169.9 (‑CH_2_-**C**ONH-), 137.5 (Ar‑**C^1^**), 137.4 (Ar‑**C^4^**), 126.9 (Ar‑**C^3/3‘^**), 119.0 (Ar‑**C^2/2‘^**), 83.7 (‑**C**≡CH), 71.3 (‑C≡**C**H), 62.6 (Ar-C^4^-**C**H_2_-), 59.9 (Pro-**C^α^**H), 58.9 (Val-**C^α^**H), 47.2 (Asn-**C^α^**H), 46.8 (Pro-**C^5^**H_2_), 37.3 (Asn-**C^β^**H_2_), 33.7 (HNCO-**C**H_2_-), 30.0 (Val-**C^β^**H), 29.5 (Pro-**C^β^**H_2_), 24.1 (Pro-**C^4^**H_2_), 19.2 (Val‑C^β^(**C**H_3_)^A^(CH_3_)^B^), 18.5 (Val-C^β^(CH_3_)^A^(**C**H_3_)^B^), 14.1 (-**C**H_2_-C≡CH).

#### 1.3.3.5 4-((*S*)-3-methyl-2-((*S*)-1-(pent-4-ynoyl-l-asparaginyl)pyrrolidine-2-carboxamido) butanamido)benzyl (4-nitrophenyl) carbonate (18a)

Under Ar-atmosphere Pentynoyl-Asn-Pro-Val-PAB-OH **17a** (44.0 mg, 86.0 µmol, 1.0 eq.) and Bis(4‑nitrophenyl) carbonate (78.0 mg, 257 µmol, 3.0 eq.) were dissolved in dry DMF (1 mL). DIPEA (73.0 µL, 428 µmol, 5.0 eq.) was added and the solution stirred for 24 h at RT until complete conversion determined by LCMS. A mixture of ACN/H_2_O (1:1, v:v, 500 µL) was added and the solution purified by RP-HPLC. Freeze-drying yielded Pentynoyl-Asn-Pro-Val-PAB-PNP-OH **18a** (49.0 mg, 72.2 µmol, 88 %) as colorless voluminous solid.

M: 678.70 g/mol | Yield: 88 %, 49.0 mg, 72.2 µmol | C_33_H_38_N_6_O_10_

**HRMS** (ESI+):	*m*/*z* (experimental):	701.2546,
*m*/*z* (calculated):	701.2542, [M+Na]^+^

^1^**H-NMR** (600 MHz, DMSO–d_6_):

δ [ppm] = 9.58 (s, 1H, Val-C^α^-CON**H**-), 8.34 (d, ^3^*J* = 7.9 Hz, 1H, Asn-N**H**), 8.31 (AA'BB', ^3^*J*_AB_ = 9.2 Hz, 2H, NO_2_-Ar-C^3/3‘^**H**-), 7.87 (d, ^3^*J* = 8.6 Hz, 1H, Val-N**H**), 7.70 (d, ^3^*J* = 8.6 Hz, 2H, ‑NH‑Ar-C^2/2‘^**H**-), 7.57 (AA'BB', ^3^*J* = 9.2 Hz, 2H, NO_2_-Ar-C^2/2‘^**H**-), 7.50 (br s, 1H, Asn-N**H**/H‘), 7.41 (d, ^3^*J* = 8.6 Hz, 2H, ‑NH‑Ar-C^3/3‘^**H**-), 6.99 (br s, 1H, Asn-NH/**H‘**), 5.25 (s, 2H, -OCH_2_-), 4.85 (td, ^3^*J* = 8.1 Hz, ^3^*J* = 6.0 Hz, 1H, Asn-C^α^**H**), 4.37 (dd, ^3^*J* = 8.4 Hz, ^3^*J* = 3.1 Hz, 1H, Pro-C^α^**H**), 4.19 (dd, ^3^*J* = 8.6 Hz, ^3^*J* = 7.2 Hz, 1H, Val-C^α^**H**), 3.84 – 3.73 (m, 1H, Pro-C^5^**H**/H‘), 3.73 – 3.67 (m, 1H, Pro-C^5^H/**H‘**), 2.76 (t, ^4^*J* = 2.5 Hz, 1H, ‑C≡C**H**), 2.65 (dd, ^2^*J* = 15.4 Hz, ^3^*J* = 8.4 Hz, 1H, Asn-C^β^**H**/H‘), 2.42 – 2.27 (m, 5H, Asn-C^β^H/**H‘**, ‑C**H_2_**‑C**H_2_**-C≡CH), 2.19 – 2.11 (m, 1H, Val-C^β^**H**), 2.11 – 2.01 (m, 1H, Pro-C^β^**H**/H‘), 1.97 – 1.81 (m, 3H, Asn-C^β^H/**H‘**, Pro-C^4^**H_2_**), 0.92 (d, ^3^*J* = 6.4 Hz, 3H, Val-C^β^(C**H_3_**)^A^(CH_3_)^B^), 0.90 (d, ^3^*J* = 6.4 Hz, 3H, Val-C^β^(CH_3_)^A^(C**H_3_**)^B^).

^13^**C**{^1^**H**}**-NMR** (151 MHz, DMSO–d_6_):

δ [ppm] = 171.8 (Asn-**C**ONH_2_), 171.4 (Pro-**C**=O), 170.7 (Asn-**C**=0), 170.0 (-CH_2_-**C**ONH-), 170.0 (Ar-C^1^-NH**C**O-), 155.3 (-OCOO**C**-), 152.0 (-OCH_2_-**C**-), 145.2 (NO_2_-**C**-), 139.2 (-NH-**C**-), 129.5 (-O**C**OO‑), 129.4 (-NH-Ar**C^3/3’^**), 125.4 (NO_2_-Ar**C^3/3’^**), 122.6 (NO_2_-Ar**C^2/2’^**), 119.2 (-NH-Ar**C^2/2’^**), 83.6 (‑**C**≡CH), 71.3 (‑C≡**C**H), 70.3 (-O**C**H_2_-), 60.0 (Pro-**C^α^**), 59.0 (Val-**C^α^**), 47.4 (Asn-**C^α^**), 47.1 (Pro-**C^5^**), 37.4 (Asn-**C^β^**), 33.7 (‑**C**H_2_‑CH_2_-C≡CH), 29.8 (Val-**C^β^**), 29.3 (Pro-**C^β^**), 24.3 (Pro-**C^4^**), 19.4 (Val‑C^β^(**C**H_3_)^A^(CH_3_)^B^), 18.6 (Val‑C^β^(CH_3_)^A^(**C**H_3_)^B^), 14.1 (‑CH_2_‑**C**H_2_-C≡CH).

#### 1.3.3.6 4-((*R*)-3-methyl-2-((*S*)-1-(pent-4-ynoyl-l-asparaginyl)pyrrolidine-2-carboxamido) butanamido)benzyl (4-nitrophenyl) carbonate (18b)

Under Ar-atmosphere Pentynoyl-Asn-Pro-d-Val-PAB-OH **17b** (64.0 mg, 125 µmol, 1.0 eq.) and Bis(4‑nitrophenyl) carbonate (121 mg, 398 µmol, 3.2 eq.) were dissolved in dry DMF (1 mL). DIPEA (106 µL, 428 µmol, 5.0 eq.) was added and the solution stirred for 24 h at RT until complete conversion determined by LCMS. A mixture of ACN/H_2_O (1:1, v:v, 500 µL) was added and the solution purified by RP-HPLC. Freeze-drying yielded Pentynoyl-Asn-Pro-d-Val-PAB-PNP-OH **18b** (68.0 mg, 100 µmol, 84 %) as colorless voluminous solid.

M: 678.70 g/mol | Yield: 84 %, 68.0 mg, 100 µmol | C_33_H_38_N_6_O_10_

**HRMS** (ESI+):	*m*/*z* (experimental):	701.2550,
*m*/*z* (calculated):	701.2542, [M+Na]^+^

^1^**H-NMR** (600 MHz, DMSO–d_6_):

δ [ppm] = 9.99 (s, 1H, Val-C^α^-CON**H**-), 8.33 – 8.27 (m, 3H, NO_2_-Ar-C^3/3‘^**H**-, Asn-N**H**), 7.84 (d, ^3^*J* = 8.5 Hz, 1H, Val-N**H**), 7.70 (d, ^3^*J* = 8.6 Hz, 2H, ‑NH‑Ar-C^2/2‘^**H**-), 7.57 (AA'BB', ^3^*J*_AB_ = 9.2 Hz, 2H, NO_2_-Ar-C^2/2‘^**H**-), 7.47 (br s, 1H, Asn-N**H**/H‘), 7.42 (d, ^3^*J* = 8.6 Hz, 2H, ‑NH‑Ar-C^3/3‘^**H**-), 7.01 (br s, 1H, Asn-NH/**H‘**), 5.25 (s, 2H, -OCH_2_-), 4.84 (td, ^3^*J* = 8.0 Hz, ^3^*J* = 6.2 Hz, 1H, Asn-C^α^**H**), 4.35 (dd, ^3^*J* = 8.6 Hz, ^3^*J* = 3.2 Hz, 1H, Pro-C^α^**H**), 4.19 (dd, ^3^*J* = 8.2 Hz, ^3^*J* = 8.2 Hz, 1H, Val-C^α^**H**), 3.80 – 3.67 (m, 2H, Pro-C^5^**H_2_**), 2.75 (t, ^4^*J* = 2.4 Hz, 1H, ‑C≡C**H**), 2.64 (dd, ^2^*J* = 15.7 Hz, ^3^*J* = 8.1 Hz, 1H, Asn-C^β^**H**/H‘), 2.42 – 2.24 (m, 5H, Asn-C^β^**H**/H‘, ‑C**H_2_**‑C**H_2_**-C≡CH), 2.19 – 2.06 (m, 1H, Val-C^β^**H**), 2.06 – 1.95 (m, 1H, Pro-C^β^**H**/H‘), 1.95 – 1.77 (m, 3H, Pro-C^β^H/**H‘**, Pro-C^4^**H_2_**), 0.88 (d, ^3^*J* = 6.7 Hz, 3H, Val-C^β^(C**H_3_**)^A^(CH_3_)^B^), 0.84 (d, ^3^*J* = 6.6 Hz, 3H, Val-C^β^(CH_3_)^A^(C**H_3_**)^B^).

^13^**C**{^1^**H**}**-NMR** (151 MHz, DMSO–d_6_):

δ [ppm] = 171.7 (Asn-**C**ONH_2_), 171.4 (Pro-**C**=O), 170.3 (Ar-C^1^-NH**C**O-), 170.2 (Asn-**C**=O), 170.0 (‑CH_2_-**C**ONH-), 155.3 (-OCOO**C**-), 152.0 (-OCH_2_-**C**-), 145.2 (NO_2_-**C**-), 139.3 (-NH-**C**-), 129.5 (‑O**C**OO‑), 129.4 (-NH-Ar**C^3/3’^**), 125.4 (NO_2_-Ar**C^3/3’^**), 122.6 (NO_2_-Ar**C^2/2’^**), 119.2 (-NH-Ar**C^2/2’^**), 83.7 (‑**C**≡CH), 71.3 (‑C≡**C**H), 70.2 (-O**C**H_2_-), 59.9 (Pro-**C^α^**), 59.0 (Val-**C^α^**), 47.2 (Asn-**C^α^**), 46.9 (Pro-**C^5^**), 37.3 (Asn-**C^β^**), 33.7 (‑**C**H_2_‑CH_2_-C≡CH), 29.9 (Val-**C^β^**), 29.5 (Pro-**C^β^**), 24.1 (Pro-**C^4^**), 19.2 (Val‑C^β^(**C**H_3_)^A^(CH_3_)^B^), 18.5 (Val‑C^β^(CH_3_)^A^(**C**H_3_)^B^), 14.1 (‑CH_2_‑**C**H_2_-C≡CH).

### 1.3.4 Linker-Crypthophycins

#### 1.3.4.1 4-((*S*)-3-methyl-2-((*S*)-1-(pent-4-ynoyl-l-asparaginyl)pyrrolidine-2-carboxamido) butanamido)benzyl (((3*S*,10*R*,16*S*,*E*)-10-(3-chloro-4-methoxybenzyl)-6,6-dimethyl-2,5,9,12-tetraoxo-16-(((2*R*,3*R*)-3-phenyloxiran-2-yl)methyl)-1-oxa-4,8,11-triazacyclo-hexadec-13-en-3-yl)methyl)(methyl)carbamate (20a)

Under Ar-atmosphere Pentynoyl-Asn-Pro-Val-PAB-PNP-OH **18a** (16.0 mg, 24.0 µmol, 1.0 eq.) and Cryptophycin-uD(Dap,Me) **19** (18 mg, 26 µmol, 1.1 eq.) were dissolved in dry DMF (0.5 mL). To the colorless solution DIPEA (24.0 µL, 142 µmol, 6.0 eq.) was added and the yellow solution stirred for 20 h at RT until complete conversion determined by LCMS. A mixture of ACNN/H_2_O (1:1, v:v, 450 µL) was added and the solution purified by RP-HPLC. Freeze-drying yielded Linker-Cryptophycin **20a** (23.3 mg, 19.5 µmol, 81 %) as colorless voluminous solid.

M: 1194.78 g/mol | Yield: 81 %, 23.3 mg, 19.5 µmol | C_61_H_76_ClN_9_O_14_

**HPLC-MS** (ESI+):	*m*/*z* (experimental):	1194.2282, t_R_ = 8.0 min.
*m*/*z* (calculated):	1194.5273, [M+H]^+^

**HRMS** (ESI+):	*m*/*z* (experimental):	1194.5245,
*m*/*z* (calculated):	1194.5273, [M+H]^+^

#### 1.3.4.2 4-((R)-3-methyl-2-((S)-1-(pent-4-ynoyl-l-asparaginyl)pyrrolidine-2-carboxamido) butanamido)benzyl (((3S,10R,16S,E)-10-(3-chloro-4-methoxybenzyl)-6,6-dimethyl-2,5,9,12-tetraoxo-16-(((2R,3R)-3-phenyloxiran-2-yl)methyl)-1-oxa-4,8,11-triazacyclo-hexadec-13-en-3-yl)methyl)(methyl)carbamate (20b)

Under Ar-atmosphere Pentynoyl-Asn-Pro-d-Val-PAB-PNP-OH **18b** (8.00 mg, 12.0 µmol, 1.0 eq.) and Cryptophycin-uD(Dap,Me) **19** (9.00 mg, 13 µmol, 1.1 eq.) were dissolved in dry DMF (0.5 mL). To the colorless solution DIPEA (12.0 µL, 71.0 µmol, 6.0 eq.) was added and the yellow solution stirred for 20 h at RT until complete conversion determined by LCMS. A mixture of ACNN/H_2_O (1:1, v:v, 450 µL) was added and the solution purified by RP-HPLC. Freeze-drying yielded Linker-Cryptophycin **20b** (13.1 mg, 11.0 µmol, 92 %) as colorless voluminous solid.

M: 1194.78 g/mol | Yield: 92 %, 13.1 mg, 11.0 µmol | C_61_H_76_ClN_9_O_14_

**HPLC-MS** (ESI+):	*m*/*z* (experimental):	1194.1378, t_R_ = 8.8 min.
*m*/*z* (calculated):	1194.5273, [M+H]^+^

**HRMS** (ESI+):	*m*/*z* (experimental):	1216,5092
*m*/*z* (calculated):	1216.5093, [M+Na]^+^

### 1.3.5 SMDCs

#### 1.3.5.1 RGD-mimetic(L)-Asn-Pro-Val-Cryptophycin-uD(Dap,Me) (21-1)

Under Ar-atmosphere Linker-Cryptophycin **20a** (6.98 mg, 5.84 µmol, 1.0 eq.) and RGD-mimetic **15a** (5.00 mg, 5.82 µmol, 1.0 eq.) were dissolved in DMF (480 µL) and Milli-Q H_2_O (320 µL). The solution was degassed by *freeze-pump-thaw* and a small amount of CuPF_6_(ACN)_4_ and THPTA fitting inside the tip of a glass pipette were added. The resulting pale-blue solution was stirred for 24 h at RT until complete conversion determined by LCMS. Purification by RP-HPLC and freeze-drying yielded SMDC **21-1** (11.0 mg, 5.36 µmol, 92 %) as colorless voluminous solid.

M: 2052.85 g/mol | Yield: 92 %, 11.0 mg, 5.36 µmol | C_102_H_139_ClN_18_O_23_S

**HPLC-MS** (ESI+):	*m*/*z* (experimental):	1026.6984, t_R_ = 7.6 min.
*m*/*z* (calculated):	1026.4907, [M+2H]^2+^

**HRMS** (ESI+):	*m*/*z* (experimental):	2051.9692,
*m*/*z* (calculated):	2051.9742, [M+H]^+^

#### 1.3.5.2 RGD-mimetic(L)-Asn-Pro-d-Val-Cryptophycin-uD(Dap,Me) (21-2)

Under Ar-atmosphere Linker-Cryptophycin **20b** (7.00 mg, 5.86 µmol, 1.0 eq.) and RGD-mimetic **15a** (5.00 mg, 5.82 µmol, 1.0 eq.) were dissolved in DMF (480 µL) and Milli-Q H_2_O (320 µL). The solution was degassed by *freeze-pump-thaw* and a small amount of CuPF_6_(ACN)_4_ and THPTA fitting inside the tip of a glass pipette were added. The resulting pale-blue solution was stirred for 24 h at RT until complete conversion determined by LCMS. Purification by RP-HPLC and freeze-drying yielded SMDC **21-2** (9.99 mg, 4.86 µmol, 84 %) as colorless voluminous solid.

M: 2052.85 g/mol | Yield: 84 %, 9.99 mg, 4.86 µmol | C_102_H_139_ClN_18_O_23_S

**HPLC-MS** (ESI+):	*m*/*z* (experimental):	1026.6990, t_R_ = 7.6 min.
*m*/*z* (calculated):	1026.4907, [M+2H]^2+^

**HRMS** (ESI+):	*m*/*z* (experimental):	2051.9739,
*m*/*z* (calculated):	2051.9742, [M+H]^+^

#### 1.3.5.3 RGD-mimetic(D)-Asn-Pro-Val-Cryptophycin-uD(Dap,Me) (21-3)

Under Ar-atmosphere Linker-Cryptophycin **20a** (7.02 mg, 5.87 µmol, 1.0 eq.) and RGD-mimetic **15b** (5.00 mg, 5.82 µmol, 1.0 eq.) were dissolved in DMF (480 µL) and Milli-Q H_2_O (320 µL). The solution was degassed by *freeze-pump-thaw* and a small amount of CuPF_6_(ACN)_4_ and THPTA fitting inside the tip of a glass pipette were added. The resulting pale-blue solution was stirred for 24 h at RT until complete conversion determined by LCMS. Purification by RP-HPLC and freeze-drying yielded SMDC **21-3** (10.6 mg, 5.16 µmol, 89 %) as colorless voluminous solid.

M: 2052.85 g/mol | Yield: 89 %, 10.6 mg, 5.16 µmol | C_102_H_139_ClN_18_O_23_S

**HPLC-MS** (ESI+):	*m*/*z* (experimental):	1026.6991, t_R_ = 7.6 min.
*m*/*z* (calculated):	1026.4907, [M+2H]^2+^

**HRMS** (ESI+):	*m*/*z* (experimental):	2051.9697,
*m*/*z* (calculated):	2051.9742, [M+H]^+^

# 2 Literature

[1]	T. G. Kapp, F. Rechenmacher, S. Neubauer, O. V. Maltsev, E. A. Cavalcanti-Adam, R. Zarka, U. Reuning, J. Notni, H.-J. Wester, C. Mas-Moruno et al., *Sci. Rep.* **2017,** *7,* 39805.

[2]	Lerchen, H.-G.; Stelte-Ludwig, B.; Heroult, M.; Zubov, D.; Gericke, K.M.; Wong, H.; Frigault, M.M.; Johnson, A.J.; Izumi, R.; Hamdy, *Cancers* **2023,** *15,* 4381.

# 3 Appendix

## 3.1 List of abbreviations

| ADDP | 1,1’-(Azodicarbonyl)dipiperidine |
| --- | --- |
| Alloc | Allyloxycarbonyl |
| aq. | aqueous |
| Boc | tert-Butyloxycarbonyl |
| br | broad |
| Cbz | Carboxybenzyl |
| DCM | Dichloromethane |
| DMF | N,N’-Dimethylformamide |
| DMSO | Dimethylsulfoxide |
| ELSD | Evaporative light scattering detector |
| ESI | Electrospray Ionization |
| eq. | equivalent |
| h | hours |
| HATU | 1-[Bis(dimethylamino)methylene]-1H-1,2,3-triazolo[4,5-b]pyridinium 3-oxide hexafluorophosphate |
| HOBt | 1-Hydroxybenzotriazol |
| HPLC | High Performance Liquid Chromatography |
| LC | Liquid Chromatography |
| min | minutes |
| MP | Medium Performance |
| MS | Mass Spectrometry |
| NMR | Nuclear Magnetic Resonance |
| quant. | Quantitative |
| rot. | rotamer |
| RP | Reverse Phase |
| TBAI | Tetrabutylammonium Iodide |
| TFA | Trifluoracetic acid |
| THF | Tetrahydrofuran |
| TLC | Thin-layer Chromatography |
| TOF | Time-of-Flight |
| UHPLC | Ultra-High Performance Liquid Chromatography |
| UV | Ultraviolet |
| v:v | Volumetric ratio |
| vis | visible |
| + | Positive polarity (MS) |

## 3.2 Spectra

**Figure S9:** ^1^H NMR spectrum (500 MHz, methanol-*d*_4_) of a mixture of aryl iodide **2a**, disubstituted aryl iodide (*), l-tyrosine (†) and residual acetone (^).

^

†

*****

**H_2_O**

†

*****

**CHD_2_OD**

 **Figure S10:** ^1^H NMR spectrum (500 MHz, methanol-*d*_4_) of a mixture of aryl iodide **2b**, disubstituted aryl iodide (*), and d-tyrosine (†).

*****

*****

†

**CHD_2_OD**

**H_2_O**

**CHD_2_OD**

*****

†

†

*****

**Figure S11:** ^1^H NMR spectrum (500 MHz, methanol-*d*_4_) of Boc-protected methyl ester **3a**.

**H_2_O**

**CHD_2_OD**

**Figure S12:** ^13^C{^1^H} NMR spectrum (126 MHz {500 MHz}, methanol-*d*_4_) of Boc-protected methyl ester **3a**.

**CD_3_OD**

 **Figure S13:** ^1^H NMR spectrum (500 MHz, methanol-*d*_4_) of Boc-protected methyl ester **3b**.

**H_2_O**

**CHD_2_OD**

 **Figure S14:** ^13^C{^1^H} NMR spectrum (126 MHz {500 MHz}, methanol-*d*_4_) of Boc-protected methyl ester **3b**.**Figure S15:** ^1^H NMR spectrum (500 MHz, chloroform-*d*) of Cbz-protected amine **5**.

**CD_3_OD**

**CHCl_3_**

**Figure S16:** ^13^C{^1^H} NMR spectrum (126 MHz {500 MHz}, chloroform-*d*) of Cbz-protected amine **5**.

**CDCl_3_**

**Figure S17:** ^1^H NMR spectrum (500 MHz, chloroform-*d*) of alkyl iodide **6**.

**CHCl_3_**

**H_2_O**

 **Figure S18:** ^1^H NMR spectrum (500 MHz, methanol-*d*_4_) of Negishi product **7a**.

**H_2_O**

**CHD_2_OD**

 **Figure S19:** ^13^C{^1^H} NMR spectrum (126 MHz {500 MHz}, methanol-*d*_4_) of Negishi product **7a**. **Figure S20:** ^1^H NMR spectrum (500 MHz, chloroform-*d*) of Negishi product **7b**.

**CHCl_3_**

**CD_3_OD**

**CDCl_3_**

**Figure S21:** ^13^C{^1^H} NMR spectrum (126 MHz {500 MHz}, chloroform-*d*) of Negishi product **7b**.

**Figure S22:** ^1^H NMR spectrum (500 MHz, chloroform-*d*) of Alloc-protected amine **8**.

**CHCl_3_**

**Figure S23:** ^13^C{^1^H} NMR spectrum (126 MHz {500 MHz}, chloroform-*d*) of Alloc-protected amine **8**.

**CDCl_3_**

 **Figure S24:** ^1^H NMR spectrum (500 MHz, methanol-*d*_4_) of Mitsunobu product **9a**.

**H_2_O**

**CHD_2_OD**

 **Figure S25:** ^13^C{^1^H} NMR spectrum (126 MHz {500 MHz}, methanol-*d*_4_) of Mitsunobu product **9a**.

**CD_3_OD**

 **Figure S26:** ^1^H NMR spectrum (500 MHz, methanol-*d*_4_) of Mitsunobu product **9b**.

**H_2_O**

**CHD_2_OD**

 **Figure S27:** ^13^C{^1^H} NMR spectrum (126 MHz {500 MHz}, methanol-*d*_4_) of Mitsunobu product **9b**.

**CD_3_OD**

 **Figure S28:** ^1^H NMR spectrum (500 MHz, methanol-*d*_4_) of sulfonamide **10a**.

**H_2_O**

**CHD_2_OD**

 **Figure S29:** ^13^C{^1^H} NMR spectrum (126 MHz {500 MHz}, methanol-*d*_4_) of sulfonamide **10a**.

**CD_3_OD**

 **Figure S30:** ^1^H NMR spectrum (600 MHz, methanol-*d*_4_) of sulfonamide **10b**.

**H_2_O**

**CHD_2_OD**

**CD_3_OD**

**Figure S31:** ^13^C{^1^H} NMR spectrum (151 MHz {600 MHz}, methanol-*d*_4_) of sulfonamide **10b**.

 **Figure S32:** ^1^H NMR spectrum (500 MHz, chloroform-*d*) of a mixture of azido amine **12**, residual DMF (†) and PPh_3_ (*).

*

†

†

**CHCl_3_**

 **Figure S33:** ^1^H NMR spectrum (500 MHz, chloroform-*d*) of a mixture of azido acid **13** and residual DCM (†).

**CHCl_3_**

†

 **Figure S34:** ^1^H NMR spectrum (500 MHz, DMSO-*d*_6_) of conjugable RGD-mimetic precursor **14a**.

**DMSO-*d*_6_**

 **Figure S35:** ^1^H NMR spectrum (600 MHz, methanol-*d*_4_) of conjugable RGD-mimetic precursor **14b**.

**CHD_2_OD**

**H_2_O**

 **Figure S36:** ^13^C{^1^H} NMR spectrum (151 MHz {600 MHz}, methanol-*d*_4_) of conjugable RGD-mimetic precursor **14b**.

**CD_3_OD**

 **Figure S37:** ^1^H NMR spectrum (600 MHz, methanol-*d*_4_) of conjugable RGD-mimetic **15a** and residual acetonitrile (*).

*****

**DMSO-*d*_6_**

**Figure S38:** ^13^C{^1^H} NMR spectrum (151 MHz {600 MHz}, methanol-*d*_4_) of conjugable RGD-mimetic **15a** and residual acetonitrile (*).

*****

*****

**DMSO-*d*_6_**

 **Figure S39:** UHPLC-MS analysis of RGD-mimetic **15a** with detection at 220 nm and associated mass spectrum (ESI, +).

**Figure S40:** ^1^H NMR spectrum (600 MHz, methanol-*d*_4_) of conjugable RGD-mimetic **15b**.

**H_2_O**

**CHD_2_OD**

**Figure S41:** ^13^C{^1^H} NMR spectrum (151 MHz {600 MHz}, methanol-*d*_4_) of conjugable RGD-mimetic **15b**.

**CD_3_OD**

 **Figure S42:** UHPLC-MS analysis of RGD-mimetic **15b** with detection at 220 nm and associated mass spectrum (ESI, +).

**Figure S43:** ^1^H NMR spectrum (600 MHz, DMSO-*d*_6_) of Pentynoyl-Asn-Pro-Val-OH **16a**. Rotamers were observed in a molar ratio of 90:10.

**DMSO-*d*_6_**

**Figure S44:** ^13^C{^1^H} NMR spectrum (151 MHz {600 MHz}, DMSO-*d*_6_) of Pentynoyl-Asn-Pro-Val-OH **16a** and residual TFA (*).

*****

*****

**DMSO-*d*_6_**

**Figure S45:** ^1^H NMR spectrum (600 MHz, DMSO-*d*_6_) of Pentynoyl-Asn-Pro-d-Val-OH **16b**. Rotamers were observed in a molar ratio of 95:5.

**DMSO-*d*_6_**

**Figure S46:** ^13^C{^1^H} NMR spectrum (151 MHz {600 MHz}, DMSO-*d*_6_) of Pentynoyl-Asn-Pro-d-Val-OH **16b** and residual TFA (*).

*****

**DMSO-*d*_6_**

**Figure S47:** ^1^H NMR spectrum (600 MHz, DMSO-*d*_6_) of Pentynoyl-Asn-Pro-Val-PAB-OH **17a**.

**DMSO-*d*_6_**

**Figure S48:** ^13^C{^1^H} NMR spectrum (151 MHz {600 MHz}, DMSO-*d*_6_) of Pentynoyl-Asn-Pro-Val-PAB-OH **17a**.

**DMSO-*d*_6_**

**Figure S49:** ^1^H NMR spectrum (600 MHz, DMSO-*d*_6_) of Pentynoyl-Asn-Pro-d-Val-PAB-OH **17b**.

**DMSO-*d*_6_**

**Figure S50:** ^13^C{^1^H} NMR spectrum (151 MHz {600 MHz}, DMSO-*d*_6_) of Pentynoyl-Asn-Pro-d-Val-PAB-OH **17b**.

**DMSO-*d*_6_**

**Figure S51:** ^1^H NMR spectrum (600 MHz, DMSO-*d*_6_) of Pentynoyl-Asn-Pro-Val-PAB-PNP **18a**.

**DMSO-*d*_6_**

**Figure S52:** ^13^C{^1^H} NMR spectrum (151 MHz {600 MHz}, DMSO-*d*_6_) of Pentynoyl-Asn-Pro-Val-PAB-PNP **18a** and residual TFA (*).

*****

**DMSO-*d*_6_**

**Figure S53:** ^1^H NMR spectrum (600 MHz, DMSO-*d*_6_) of Pentynoyl-Asn-Pro-d-Val-PAB-PNP **18b**.

**DMSO-*d*_6_**

**Figure S54:** ^13^C{^1^H} NMR spectrum (151 MHz {600 MHz}, DMSO-*d*_6_) of Pentynoyl-Asn-Pro-d-Val-PAB-PNP **18b** and residual TFA (*).

*****

**DMSO-*d*_6_**

 **Figure S55:** UHPLC-MS analysis of SMDC **21-1** with detection at 220 nm and associated mass spectrum (ESI, +).

**Figure S56:** UHPLC-MS analysis of SMDC **21-2** with detection at 220 nm and associated mass spectrum (ESI, +).

 **Figure S57:** UHPLC-MS analysis of SMDC **21-3** with detection at 220 nm and associated mass spectrum (ESI, +).

**Figure S58:** ^1^H NMR spectrum (500 MHz, methanol-*d*_4_) of a mixture of methyl ester **S1a**, diiodo l‑tyrosine methyl ester (*), and l-tyrosine methyl ester (†).

*****

†

**CHD_2_OD**

*****

†

**H_2_O**

**Figure S59:** ^1^H NMR spectrum (500 MHz, methanol-*d*_4_) of a mixture of methyl ester **S1b**, diiodo d‑tyrosine methyl ester (*), d-tyrosine methyl ester (†) and residual methanol (C**H_3_**OH).

**H_2_O**

*****

*****

†

†

**CH_3_OH**

**CHD_2_OD**

**Figure S60:** ^1^H NMR spectrum (500 MHz, chloroform-*d*) of a mixture of tosylated product **S2**, residual DCM (†) and H_2_O (*).

*

†

**CHCl_3_**

**Figure S61:** ^13^C{^1^H} NMR spectrum (126 MHz {500 MHz}, chloroform-*d*) of tosylated product **S2**.

**CDCl_3_**

**Figure S62:** ^1^H NMR spectrum (500 MHz, chloroform-*d*) of a mixture of azide **S3**, residual DMF (†) and H_2_O (*).

*

†

†

†

**CHCl_3_**
